# The effect of pre-pregnancy body mass index on breastfeeding initiation, intention and duration: A systematic review and dose-response meta-analysis

**DOI:** 10.1016/j.heliyon.2020.e05622

**Published:** 2020-12-07

**Authors:** Seyed-Saeed Hashemi-Nazari, Jalil Hasani, Neda Izadi, Farid Najafi, Jamal Rahmani, Parisa Naseri, Abdolhalim Rajabi, Cain Clark

**Affiliations:** a Prevention of Cardiovascular Disease Research Center, Department of Epidemiology, School of Public Health and Safety, Shahid Beheshti University of Medical Sciences, Tehran, Iran; b Torbat Jam Faculty of Medical Sciences, Torbat Jam, Iran; c Student Research Committee, Department of Epidemiology, School of Public Health and Safety, Shahid Beheshti University of Medical Sciences, Tehran, Iran; d Department of Epidemiology, Research Center for Environmental Determinants of Health (RCEDH), Kermanshah University of Medical Sciences, Kermanshah, Iran; e Department of Community Nutrition, Faculty of Nutrition and Food Technology, National Nutrition and Food Technology Research Institute, Shahid Beheshti University of Medical Sciences, Tehran, Iran; f Research Center for Social Determinants of Health, Research Institute for Endocrine Sciences, Shahid Beheshti University of Medical Sciences, Tehran, Iran; g Environmental Health Research Center, Faculty of Health, Golestan University of Medical Sciences, Gorgan, Iran; h Centre for Sport, Exercise and Life Sciences, Coventry University, Coventry, CV1 5FB, United Kingdom

**Keywords:** Public health, Women's health, Reproductive medicine, Obstetrics and gynecology, Reproductive system, Clinical research, Pre-pregnancy, Body mass index, Breastfeeding, Initiation, Duration

## Abstract

Overweight and obesity not only are major risk factors for number of chronic diseases, but also a risk factor for pregnancy complications in women. The present study aims to investigate the association between pre-pregnancy BMI and the persistence and duration of BF. The electronic databases including Medline (PubMed), Scopus, Embase, Web of Science and Google Scholar were searched for papers with titles and/or abstracts including one of our keywords and published up to 15 April 2019. For dose-response relationship, the two-stage random-effects meta-analysis was performed using the “dosresmeta” function in R software. Thirty-two studies with the effect of pre-pregnancy BMI on BF initiation, intention and duration were included in the present study. Based on crude and adjusted OR models, the risk of BF cessation increased by 4% (OR = 1.04; 95% CI: 1.02–1.05) with an increase in a unit of BMI. In addition, based on crude and adjusted RR models, the risk of BF cessation increases by 2% and 1% (crude RR = 1.02; 95% CI: 1.01–1.03 and adjusted RR = 1.01; 95% CI: 0.99–1.02) with an increase in one unit of BMI. Based on the result, the health care professionals and other key stakeholders should be aware of the impact excess weight, and that women who are overweight or obese should be encouraged with continued access to guidance, counseling and support, starting from conception, to maximize BF outcomes.

## 1 Introduction

Overweight and obesity are defined as abnormal or excess fat accumulation, and are a risk for general health [1]. Given that the prevalence of obesity has increased, universally, it has become a global public health concern [2]. The prevalence of overweight and obesity has almost tripled between 1975 and 2016 and has reached an epidemic in the world. According to the world health organization (WHO) in 2016, more than 1.9 billion adults were overweight and 650 million were obese. At least 2.8 million people each year die as a result of being overweight or obesity [3]. In the past, obesity was associated with high-income countries, however, it is now also prevalent in both low- and middle-income countries [4].

Overweight and obesity not only are major risk factors for the number of chronic diseases, including diabetes, cardiovascular diseases, and cancer, but also a risk factor for pregnancy complications in women [2]. According to recent studies; hypertension, preeclampsia, gestational diabetes, cesarean section, long delivery, intrauterine fetal death and congenital anomalies in obese mothers are more common than others [5,6]. Women with high body mass index (BMI) have delays in establishing lactation after giving birth. In addition, overweight and obesity increase the risk of early cessation of breastfeeding (BF) [7]. In fact, BF can reduce the risk of infectious disease and overweight and obesity among children [8]. The World Health Organization (WHO) recommends that exclusive/full breastfeeding (EBF) should continue to 6 months of age, with the gradual addition of complementary foods until 24 months of age [1]. Worldwide, about 45% of infants are breastfed within the first hour after birth, and data from 2011 to 2017 indicate that only 40% of infants between 0 and 6 months of age are exclusively breastfed [9]. According to data from WHO, 39% of women aged older than 18 years old were overweight around the world in 2016 [1,10]. Studies show that women with pre-pregnancy underweight, overweight, and obesity, are less likely to initiate breastfeeding and/or continue EBF and/or any breastfeeding (ABF) to recommended time [11, 12, 13, 14].

In current systematic reviews, women with pre-pregnancy overweight and obesity were less likely to initiate BF and continue with EBF. However, antecedent evidence does not consider a dose-response association between maternal BMI and EBF/ABF [8,15, 16, 17, 18]. Therefore, in the present study, the association between pre-pregnancy body mass index and the persistence and duration of BF will be investigated in a systematic review and dose-response meta-analyses.

## 2 Methods

### 2.1 Search strategy

Five electronic databases including Medline (PubMed), Scopus, Embase, Web of Science and Google Scholar were searched for papers with titles and/or abstracts including one of our keywords and published up to 15 April 2019. Different combinations of general keywords, MeSH and Emtree terms were: “BMI”, “Body Mass Index”, “Overweight”, “Obesity”, “Breast Feeding”, “Breast Feeding Initiation”, “Breast Feeding Intensity”, “Breast Feeding Duration”, “Cross-Sectional Studies”, “Cohort Studies”, “Case-Control Studies”, “Prospective Studies”, and “Retrospective Studies” (Appendix 1). Duplicate papers were checked and deleted. In addition, if the full-text of paper were unavailable, we requested the paper from the author via email. We used the abstracts of paper (if there were enough information and data) or papers were excluded from the study where the author did not answer.

### 2.2 Study selection

All English observational studies on pre-pregnancy BMI and breastfeeding initiation, intention and duration were included. The title and abstract of publications were screened.

The criteria for inclusion of studies in the meta-analysis were:

1. The study was included if enough information about pre-pregnancy BMI before significant weight gain was available. Therefore, all studies in which there was a record of first BMI within the first trimester or earlier, either self-reported, by interview or recorded in a medical report by measurement were included.
2. Women with a pregnancy
3. Primary observational studies

In addition, we excluded studies:

4. With a report of “maternal BMI” [17,19, 20, 21].
5. Without mention of BMI type (pre-pregnancy or maternal BMI)
6. Those studies in which overweight was the reference group [22,23].
7. If they used a common data set in several studies. In these cases, a newer study with the most information was included in the analysis [13,24,25].
8. Also, the studies which had not reported the odds ratio (OR), relative risk (RR), hazard ratio (HR), confidence interval (CI) or standard error (SE), the percent or the number of initiation or intention of BF and the number of BMI in each category or inadequate data to calculate them
9. Case reports, letter to editor and previous systematic reviews or meta-analyses

### 2.3 Screening and data extraction

The relevant publications were reviewed independently by two authors using EndNote X8 software. Discrepancies between authors were resolved by consensus or if they did not reach to agreement an expert provided the final result. Then, qualified studies were obtained for full-text screening. The three authors extracted the information in order to identify eligible studies. After the final evaluation, the following information was extracted: the name of the first author, date of publication, country, sample size, study design, the number of BMI group, percent or the number of initiation and intention of BF, mean or median duration of BF, crude and adjusted OR, RR & HR and 95% confidence interval (CI). For the purpose of this study, the dose (the mean for each BMI category) was calculated based on Najafi et al, study [26], so that, doses were defined as:

BMI: 18.5–24.9 = 21.7; BMI: 25–29.9 = 27.45; BMI: 30–34.9 = 32.45; BMI: 35–39.9 = 37.45

BMI: 19.8–26 = 22.9; BMI: 20–24.9 = 22.4; BMI: 19–26 = 22.5; BMI: 26–29 = 27.5; BMI: 30–39.9 = 35; BMI: 40–49.9 = 45

BMI≤20 = 18.5; BMI<25 = 21; BMI≥25 = 30; BMI<30 = 23.7; BMI≥30 = 34.6; BMI≥35 = 38.5. Also, considering 18 for BMI≤18.5, 41 for BMI≥40 and 51 for BMI≥50.

In addition, for those studies in which there was a report for number and/or percent of initiation/intention of BF by levels of BMI, we calculated the crude OR/RR [2,11,22,27,28,29,30,31,32]. In order to calculate OR for the association between levels of BMI and BF, and if the authors used the non-initiation/intention group as reference, we calculated OR by the proportion of non-initiation/intention of BF to initiation/intention of BF (by using the reciprocal characteristic of OR) [1,7,27,33, 34, 35, 36, 37, 38, 39, 40, 41]. Also, in a study by Hilson et al. (2006) [42], we used values for adjusted ORs and RRs for a group of women “within IOM” as they placed normal-weight women (within IOM) as the reference group for all other strata and BMIs.

All procedures performed in studies involving data extracted from articles were in accordance with the ethical standards of the Shahid Beheshti University of Medical Sciences research committee and with the 1964 Helsinki declaration and its later amendments or comparable ethical standards.

### 2.4 Quality assessment of studies

The quality of included studies, was assessed using the Newcastle-Ottawa quality assessment scale for cohort, case-control and cross-sectional studies [43], based on criteria including: the type of study, sample size, participant selection, representativeness of the sample (case or expose group), adequacy of follow up, comparability and method of ascertainment for cases and controls. Finally, 40 studies with high and medium quality were included in the analyses.

### 2.5 Statistical analysis

The “metaprop” and “metan” command was performed to aggregate data from studies using Stata software (version 12) and fixed or random-effects models were applied based on the degree of heterogeneity between the studies and significance of the Cochran's Q-test or a large Higgins and Thompson's I^2^ value. The cumulative meta-analysis was performed using the “metacum” command and standard error for the prevalence of BF was calculated by the binomial distribution formula. The pooled effects of pre-pregnancy BMI levels (with 95% CI) on BF initiation, intention and duration were presented separately as odds ratio (OR), relative risk (RR) and hazard ratio (HR). In order to investigate the dose-response relationship, the two-stage random-effects meta-analysis was performed using the “dosresmeta” function in R software (version 3.5.2). In this analysis, the studies which had not reported the percent or the number of BF initiation and the number of BMI in each level were excluded then linear, quadratic and cubic spline models were used to evaluate the relationship between pre-pregnancy BMI (dose) and BF initiation. Different models were fitted to the data and the best model was chosen according to the AIC and BIC. The dose-response analysis for the duration of BF was not performed due to the low number of studies. In addition, the funnel plot, Begg's and Egger's test were applied to examine the publication bias using “metafunnel” and “metabias” command in Stata software.

## 3 Results

**Figure 1:**
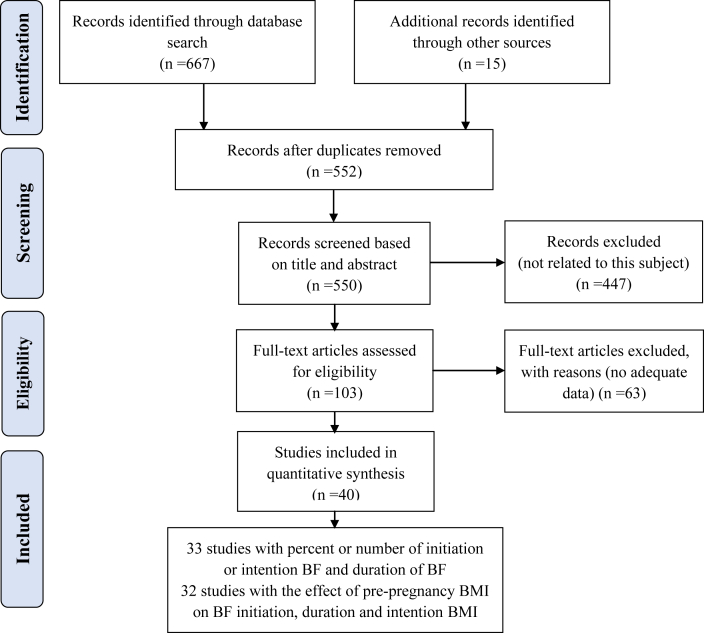
Flow diagram of the study selection process and including publications for the dose-response meta-analysis of pre-pregnancy BMI and breastfeeding initiation, intention and duration.

Consistent with standard meta-analysis techniques, the PRISMA guidelines, Up to April 2019, thirty-three studies with percent or number of initiation/intention or duration of BF and thirty-two studies with the effect of pre-pregnancy BMI on BF initiation, intention and duration were included in the present study (Figure 1). The studies were conducted in 17 countries. More details about the studies are shown in Appendix 2.

### 3.1 The overall prevalence of BF initiation/intention by pre-pregnancy BMI levels

**EBF:** The overall prevalence of EBF was 48.4, 61.6, 53.9, and 37.8% for underweight, normal, overweight and obese women, respectively (Table 1).

**Table 1:** The overall prevalence of EBF and ABF by pre-pregnancy BMI (kg/m^2^) levels.

Type of BF		Underweight	Normal	Overweight	Obesity (Class I, II, III)
EBF	Prevalence %	48.4	61.6	53.9	37.8
	95% CI	12.2–85.6	42.6–78.9	33.6–73.6	23.8–52.9
	No. of studies	6	24	20	30
ABF	Prevalence %	75.9	72.1	68.7	64.4
	95% CI	62.9–86.8	63.5–79.9	60.4–76.3	59.4–69.2
	No. of studies	3	14	8	18
Not Reported	Prevalence %	86	86.5	77.3	71.8
	95% CI	82.7–88.9	83.6–89.1	69.1–84.5	68.3–75.1
	No. of studies	9	27	28	29
EBF and/or ABF	Prevalence %	73.6	75.6	68.2	58.3
	95% CI	68.7–78.2	72.2–78.9	61.8–74.3	55.2–61.4
	No. of studies	18	65	56	77

EBF = Exclusive/Full Breastfeeding; ABF = Any Breastfeeding.

**ABF:** The overall prevalence of ABF was 75.9, 72.1, 68.7, and 64.4% for underweight, normal, overweight and obese women, respectively (Table 1).

**Not Reported:** The overall prevalence of BF in studies that did not report the type of BF was 86, 86.5, 77.3, and 71.8% for underweight, normal, overweight and obese women, respectively (Table 1).

**EBF and/or ABF:** The overall prevalence of EBF and/or ABF was 73.6, 75.6, 68.2, and 58.3% for underweight, normal, overweight and obesity, respectively (Table 1).

### 3.2 The cumulative prevalence of BF initiation/intention among women

Based on the cumulative meta-analysis, the trend of BF prevalence among underweight, normal, overweight and obese women decreased from 2001 to 2019.

### 3.3 The overall effect of pre-pregnancy BMI on the risk of BF cessation

**Crude and adjusted odds ratio:** The overall crude estimate for odds of BF cessation among underweight, overweight and obese women were 1.3, 1.37, and 1.72, respectively. The corresponding values for adjusted OR were 1.14, 1.24, and 1.47, respectively (Table 2).

**Table 2:** The overall effect of pre-pregnancy BMI (kg/m^2^) on the cessation of EBF and ABF in BMI levels.

BMI Categories	Crude Odds Ratio				Adjusted Odds Ratio			
	No. of studies	OR (95% CI)	P for Heterogeneity	I^2^ (%)	No. of studies	OR (95% CI)	P for Heterogeneity	I^2^ (%)
Underweight	25	1.3^b^ (1.18–1.42)	<0.0001	83.6	9	1.14^a^ (1.12–1.17)	<0.0001	30.7
Normal	68	1	-	-	30	1	-	-
Overweight	62	1.37^b^ (1.10–1.70)	<0.0001	99.6	29	1.24^b^ (1.14–1.34)	0.001	89.7
Obesity (Class I, II, III)	77	1.72^b^ (1.56–1.89)	<0.0001	96.3	28	1.47^b^ (1.23–1.75)	<0.0001	98.4
BMI Categories	Crude Relative Risk				Adjusted Relative Risk			
	No. of studies	RR (95% CI)	P for Heterogeneity	I^2^ (%)	No. of studies	RR (95% CI)	P for Heterogeneity	I^2^ (%)

Underweight	18	1.18^b^ (1.11–1.25)	<0.0001	86.1	4	1^a^ (0.89–1.11)	0.98	39.6
Normal	55	1	-	-	6	1	-	-
Overweight	44	1.18^b^ (1.03–1.35)	<0.0001	99.6	6	1.05^a^ (1.01–1.1)	0.01	24.4
Obesity (Class I, II, III)	64	1.32^b^ (1.25–1.40)	<0.0001	96.1	4	1.18^a^ (1.03–1.36)	0.01	-
BMI Categories	Crude Hazard Ratio				Adjusted Hazard Ratio			
	No. of studies	HR (95% CI)	P for Heterogeneity	I^2^ (%)	No. of studies	HR (95% CI)	P for Heterogeneity	I^2^ (%)

Underweight	-	-	-	-	2	1^a^ (0.84–1.19)	0.99	-
Normal	-	-	-	-	6	1	-	-
Overweight	-	-	-	-	3	1.17^a^ (1.05–1.31)	0.005	-
Obesity (Class I, II, III)	-	-	-	-	5	1.69^a^ (1.43–2)	<0.001	20.8

EBF = Exclusive/Full Breastfeeding; ABF = Any Breastfeeding.

a Fixed effect model.

b Random effect model.

**Crude and adjusted relative risk:** The overall crude estimate for the risk of BF cessation among underweight, overweight and obese women were 1.18, 1.18 and 1.32, respectively. The corresponding values for adjusted RR were 1, 1.05, and 1.18, respectively (Table 2).

**Adjusted hazard ratio:** The adjusted hazard for BF cessation in underweight, overweight and obese women were 1, 1.17, and 1.69, respectively (Table 2).

### 3.4 The overall effect of pre-pregnancy BMI on the risk of short-time BF

**Crude and adjusted odds ratio:** The crude point estimate for odds of short-time BF among underweight, overweight and obese women were 0.85, 1.14, and 1.22, respectively. The corresponding values for adjusted OR were 0.84, 1.07, and 1.18, respectively (Table 3).

**Table 3:** The overall effect of pre-pregnancy BMI (kg/m^2^) on the breastfeeding duration in BMI levels.

BMI Categories	Crude Odds Ratio				Adjusted Odds Ratio			
	No. of studies	OR (95% CI)	P for Heterogeneity	I^2^ (%)	No. of studies	OR (95% CI)	P for Heterogeneity	I^2^ (%)
Underweight	1	0.85 (0.62–1.16)	0.3	-	1	0.84 (0.62–1.13)	0.25	53
Normal	4	1	-	-	4	1	-	-
Overweight	4	1.14^a^ (1.05–1.25)	0.002	-	4	1.07^a^ (0.98–1.19)	0.13	50.2
Obesity (Class I, II, III)	2	1.22^a^ (1.11–1.35)	<0.0001	43.3	2	1.18^a^ (1.06–1.32)	0.003	41.7
BMI Categories	Crude Relative Risk				Adjusted Relative Risk			
	No. of studies	RR (95% CI)	P for Heterogeneity	I^2^ (%)	No. of studies	RR (95% CI)	P for Heterogeneity	I^2^ (%)

Underweight	1	0.97 (0.81–1.15)	0.72	-	3	0.96^a^ (0.92–1.23)	0.04	12.9
Normal	5	1	-	-	7	1	-	-
Overweight	5	1.04^a^ (0.95–1.15)	0.33	-	7	1.08^a^ (1.06–1.1)	<0.001	39.1
Obesity (Class I, II, III)	5	0.95^a^ (0.85–1.06)	0.36	53.5	11	1.21^b^ (1.12–1.31)	<0.001	78
BMI Categories	Crude Hazard Ratio				Adjusted Hazard Ratio			
	No. of studies	HR (95% CI)	P for Heterogeneity	I^2^ (%)	No. of studies	HR (95% CI)	P for Heterogeneity	I^2^ (%)

Underweight	2	1.27^a^ (0.98–1.64)	0.06	-	4	1.02^a^ (0.95–1.09)	0.47	18.4
Normal	4	1	-	-	6	1	-	-
Overweight	4	1.02^a^ (0.90–1.16)	0.69	62.9	6	1.12^b^ (1.02–1.22)	<0.001	64.2
Obesity (Class I, II, III)	6	1.18^b^ (0.93–1.49)	0.003	69.9	6	1.26^b^ (1.06–1.49)	<0.001	55.9

a Fixed effect model.

b Random effect model.

**Crude and adjusted relative risk:** The crude point estimate for the risk of short-time BF among underweight, overweight and obese women were 0.97, 1.04 and 0.95, respectively. The corresponding values for adjusted RR were 0.96, 1.08, and 1.21, respectively (Table 3).

**Crude and adjusted hazard ratio:** The crude estimate of hazard for short-time BF among underweight, overweight and obese women were 1.27, 1.02 and 1.18, respectively. The corresponding values for adjusted HR were 1.02, 1.12 and 1.26, respectively (Table 3).

### 3.5 Dose-response association between pre-pregnancy BMI and risk of BF cessation

**Figure 2:**
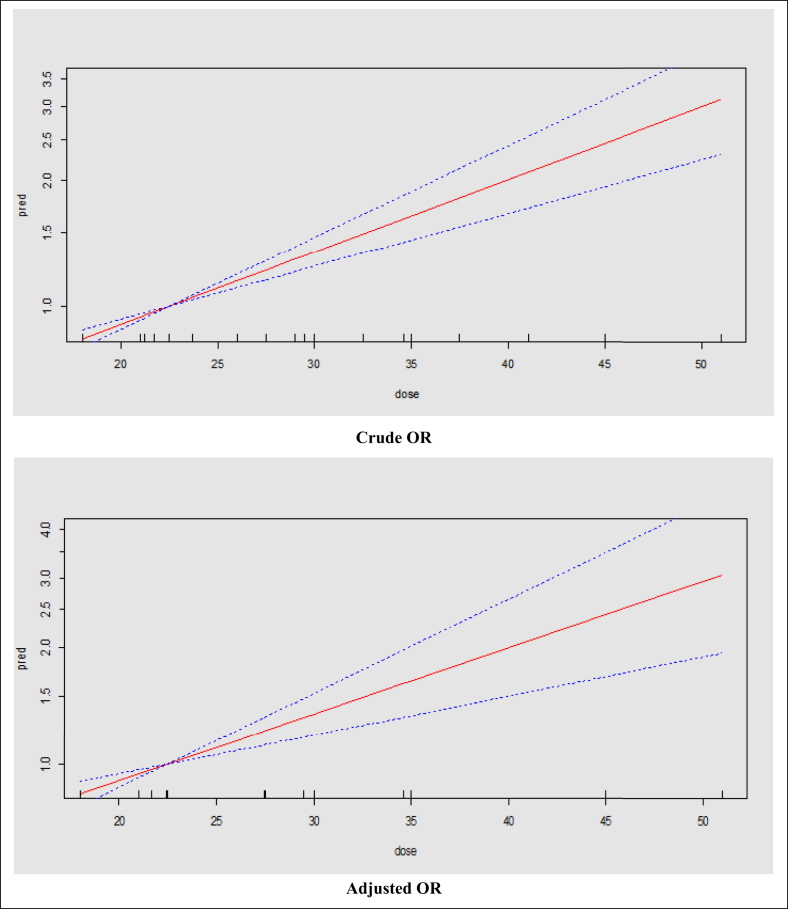
The dose-response relationship of BMI (kg/m^2^) and risk of BF cessation based on the linear model and crude and adjusted OR; the solid line represents the fitted linear trend and dash line represents the 95% confidence interval.

**Figure 3:**
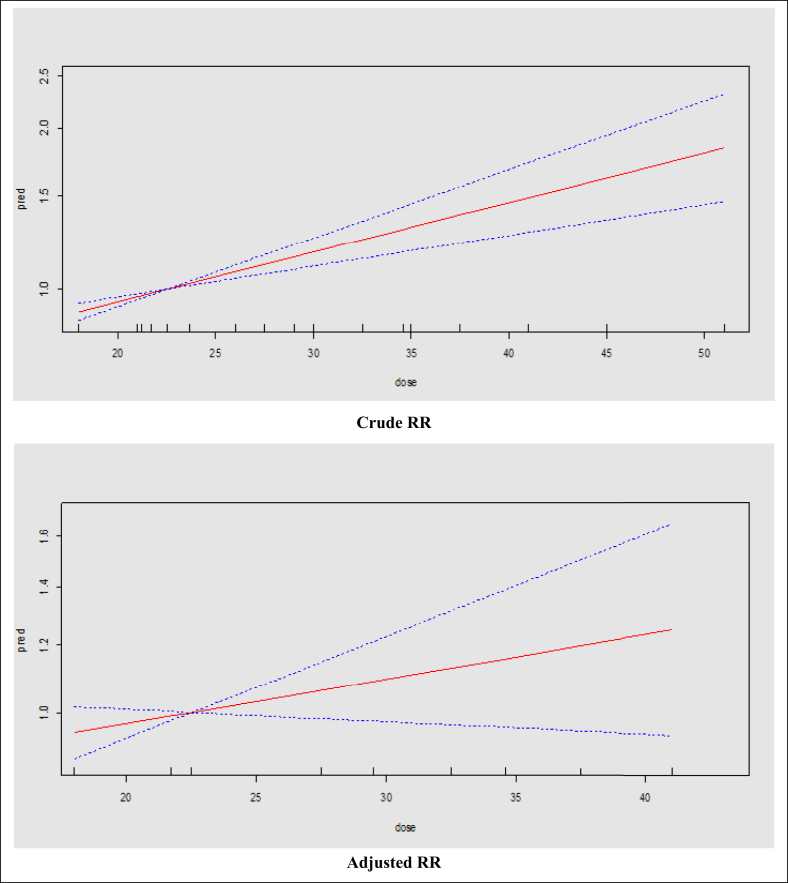
The dose-response relationship of BMI (kg/m^2^) and risk of BF cessation based on the linear model and crude and adjusted RR; the solid line represents the fitted linear trend and dash line represents the 95% confidence interval.

Using dose-response meta-analysis, there was a significant linear association between risk of BF cessation and pre-pregnancy BMI. Based on crude and adjusted OR models, the risk of BF cessation increased by 4% (OR = 1.04; 95% CI: 1.02–1.05) with an increase in a unit of BMI (Figure 2). In addition, based on crude and adjusted RR models, the risk of BF cessation increases by 2% and 1% (crude RR = 1.02; 95% CI: 1.01–1.03 and adjusted RR = 1.01; 95% CI: 0.99–1.02) with an increase in one unit of BMI (Figure 3).

### 3.6 Publication bias of risk of BF cessation in women

The Begg's and Egger's test indicates that there is no publication bias in the present study (except in the crude odds ratio of the risk of BF cessation) (p > 0.05).

## 4 Discussion

More than one-third of women, aged over 18 years old, were classified as being overweight in 2016 [1,44], with many independent bodies projecting increases, globally, in the coming years [45,46]. Empirical data exists to suggest that women with pre-pregnancy underweight, overweight, and obesity, are less probable to initiate BF, and/or continue EBF, and/or any breastfeeding (ABF) [11, 12, 13, 14]. Previously, it has been reported that women with pre-pregnancy overweight and obesity were less likely to initiate BF and continue with EBF. However, such available evidence does not consider a dose-response association between maternal BMI and EBF/ABF [8,15, 16, 17, 18]. Therefore, in the present study, we sought to investigate the association between pre-pregnancy BMI and the persistence and duration of BF in a systematic review and dose-response meta-analyses. In accordance with the aim of this study, we found that the trend of BF prevalence among underweight, normal weight, overweight and obese women decreased from 2001 to 2019, the lowest prevalence was related to overweight and obesity. Accordingly, overweight and obese women were more likely (in terms of relative risk, hazard ratio and odds ratio) to not initiate, or cease, EBF/ABF, as compared to normal-weight women. Furthermore, there was a significant linear association between risk of BF cessation and pre-pregnancy BMI, where the risk of BF cessation increased by 4% per 1-unit increase of BMI.

In a previous systematic review, Turcksin et al. (2014) reported that obese women plan to BF for a shorter period and are less likely to initiate and continue BF than normal-weight counterparts [16]; in the present study, this assertion was affirmed statistically, where we also incorporated a robust, hitherto unseen, dose-response meta-analysis. It is important to discern why adverse BF outcomes are more prevalent among overweight and obese women. One conceivable explanation relates to the axiom that women with excess weight will have larger breasts, which can act as a mechanical barrier, and therein have a negative impact on milk production, secretion and infant latching [47]. A significantly higher prevalence of obese women report difficulties BF, including cracked nipples, fatigue or difficulty initiating BF, whilst resident on the maternity ward, at 1- and 3-months post-partum, respectively, in comparison with normal-weight counterparts [28]. Concerning milk supply, in comparison to normal-weight counterparts, obese women have a significantly lower milk transfer, more likely to perceive their milk supply as insufficient, and are more likely to report feeling uncomfortable BF at 3 months in social contexts [28]. The further putative explanation for the finding that overweight and obesity is associated with negative BF outcomes includes, that excessive adipose tissue acts as a reservoir of progesterone, and this can be an obstacle to the generation and secretion of lactogenesis II [42], which influences BF initiation [48]. However, in Garcia et al. (2016) it was reported that, in the overweight women, the indicative function of body fat was limited, suggesting that overweight may only have a limited impact on breastfeeding initiation [15]; although contrastingly, we found that even being classified as overweight was associated with negative BF outcomes. Further, having excess weight is associated with a plethora of injurious comorbidities, including the history of cesarean-section delivery, gestational diabetes, and postpartum complications, which, coincidentally, are also strongly associated with poor BF outcomes [49]. In addition, increasing evidence indicates that the quantity and the quality of human milk may be, in part, modulated by body composition [50], suggesting that problems associated with BF may be entwined with diet. Meyer et al. (2017) demonstrated that maternal diet significantly alters the milk microbiome, human milk oligosaccharides (HMO) composition, and abundance of gut-associated taxa [51]. Whilst postnatally, Burchenal et al. (2017) reported that the quality of carbohydrate consumed at 2-weeks and 4-months postpartum is associated with human breast milk quality, suggesting that modifiable factors, such as diet, may deleteriously impact bioactive components [52].

This systematic review and meta-analysis highlighted that overweight and obesity have negative associations with BF. In addition, there is also strong evidence to suggest that obesity is an independent cause of pregnancy and delivery complications, before BF has commenced, conceivably influencing intentions to BF [53, 54, 55, 56]. Although there is a paucity of data available on the associated cost induced by the pregnancy of obese women, Galtier-Dereure et al [55] reported that the cost for the obese mothers was five times higher than for the normal-weight mothers. Moreover, offspring of obese mothers have a higher risk for intrauterine fetal death [57,58], congenital abnormalities [57], head trauma, shoulder dystocia, brachial plexus lesions, fractures of the clavicle [59] and increased risk of mortality within the first year [60,61]; whilst paradoxically, BF is associated with the amelioration of many of these injurious pathologies. In this systematic review and meta-analysis, a number of negative BF outcomes are identified in obese women. Findings from this review suggest that health care professionals should consider obese women at risk for poor BF success and that they merit additional attention. To optimize the BF practice in overweight and obese women, health care professionals could facilitate additional education and assistance for BF, starting before conception, and into the post-partum period. Breastfeeding promotion interventions and counseling practices targeted at overweight and obese women specifically should be developed and tested for efficacy before implementation to ensure successful initiation and continuation of BF, notwithstanding, however, such interventions require tactful planning so to avoid weight-related stigma.

### 4.1 Strengths and limitations

The strengths of this review include that it reports the synthesis of the currently available evidence, whilst concurrently providing hitherto unseen quantitative evidence, in the form of a dose-response meta-analysis, and contains a large, overall sample. Some limitations of this review also should be noted. The measurement of exposures and outcomes include self-report values, and it has been reported that women often overstate their height and underreport their weight, which could yield inaccurate BMI reports. In addition, recall bias would be generated when BF was self-reported, and as such, it is conceivable that the association could be of greater magnitude considering that the exposures may be underestimated, and may have recall bias. Furthermore, an important point that needs to be highlighted is that BF is a cultural issue that varies among countries [62], and thus, more detailed, culturally specific trials must be conducted.

### 4.2 Conclusions

Findings from the present study suggest that BF rates are lowest in women who are overweight or obese, and there is a significantly greater hazard, relative risk, and odds ratio, respectively, for overweight and obese women to not initiate, or cease, EBF/ABF. Finally, we found that there is a significant linear association between risk of BF cessation and pre-pregnancy BMI, where the risk of BF cessation increases by 4% per 1-unit increase of BMI. Clearly, the influence of excessive weight on BF is multifaceted, with psychological, physiological, emotional and mechanical barriers all, likely, having mediating roles. Health care professionals and other key stakeholders should be aware of the impact excess weight, and that women who are overweight or obese should be encouraged with continued access to guidance, counseling and support, starting from conception, to maximize BF outcomes.

## Declarations

### Author contribution statement

All authors listed have significantly contributed to the development and the writing of this article.

### Funding statement

This work was supported by the Student Research Committee, 10.13039/501100005851Shahid Beheshti University of Medical Sciences (1397/69239).

### Data availability statement

Data is included in supplementary material.

### Declaration of interests statement

The authors declare no conflict of interest.

### Additional information

No additional information is available for this paper.
