# Supplementary material for: The effect of pre-pregnancy body mass index on breastfeeding initiation, intention and duration: A systematic review and dose-response meta-analysis
Source: Heliyon. 2020 Dec 7;6(12):e05622. doi: 10.1016/j.heliyon.2020.e05622 (PMC7725724; doi:10.1016/j.heliyon.2020.e05622)
Supplement: Appendix_1 [file mmc1.docx]

**The effect of pre-pregnancy body mass index on breastfeeding initiation, intention and duration: a systematic review and dose-response meta-analysis**

**Search Strategy in Medline (PubMed):**

((("Body Mass Index"[Mesh] OR "BMI"[tiab] OR "Overweight"[Mesh] OR "Obesity"[Mesh]) AND ("Breast Feeding"[Mesh] OR "Breast Feeding Initiation"[tiab] OR "Breast Feeding Intensity"[tiab] OR "Breast Feeding Duration"[tiab]) AND ("Cross-Sectional Studies"[Mesh] OR "Cohort Studies/methods"[Mesh] OR "Case-Control Studies/methods"[Mesh] OR "Prospective Studies"[Mesh] OR "Retrospective Studies"[Mesh])

**Search Strategy in Embase:**

('body mass'/exp OR 'bmi (body mass index)' OR 'quetelet index' OR 'body ban mass' OR 'body mass' OR 'body mass index' OR 'bmi'/exp OR 'obesity'/exp OR 'adipose tissue hyperplasia' OR 'adipositas' OR 'adiposity' OR 'alimentary obesity' OR 'body weight, excess' OR 'corpulency' OR 'fat overload syndrome' OR 'nutritional obesity' OR 'obesitas' OR 'obesity' OR 'overweight') AND ('breastfeeding initiation' OR 'breastfeeding intensity' OR 'breastfeeding duration') AND ('cross-sectional study'/exp OR 'cross-sectional design' OR 'cross-sectional research' OR 'cross-sectional studies' OR 'cross-sectional study' OR 'cohort study' OR 'case control study'/exp OR 'case control study' OR 'case-control studies' OR 'case-control study' OR 'control study, case' OR 'matched case control' OR 'matched case control studies' OR 'matched case control study' OR 'prospective study'/exp OR 'prospective method' OR 'prospective studies' OR 'prospective study' OR 'study, prospective' OR 'retrospective study'/exp OR 'ex post facto design' OR 'retrospective design' OR 'retrospective panel studies' OR 'retrospective panel study' OR 'retrospective studies' OR 'retrospective study' OR 'study, retrospective' OR 'observational study'/exp OR 'non experimental studies' OR 'non experimental study' OR 'nonexperimental studies' OR 'nonexperimental study' OR 'observation studies' OR 'observation study' OR 'observational studies' OR 'observational studies as topic' OR 'observational study' OR 'observational study as topic')

**Search Strategy in Web of Science:**

#1: TS=("Body Mass Index" OR BMI OR Obesity OR Overweight)

#2: TS=("breast feeding initiation" OR "breast feeding intensity" OR "breast feeding duration ")

#3: TS=("Cross sectional" OR "Cross sectional studie*" OR Cohort OR "Cohort study" OR "Case Control studie*" OR Prospective OR Retrospective OR "Observational studie*")

Combine: #1 AND #2 AND #3

**Search Strategy in Scopus:**

TITLE-ABS-KEY ("Body Mass Index" OR "BMI" OR Obesity OR Overweight) AND TITLE-ABS-KEY ("Breast Feeding Initiation" OR "Breast Feeding Intensity" OR "Breast Feeding Duration") AND TITLE-ABS-KEY ("Cross sectional" OR "Cross sectional studie*" OR Cohort OR "Cohort study" OR "Case Control studie*" OR Prospective OR Retrospective OR "Observational studie*")
