# Supplementary material for: The effect of pre-pregnancy body mass index on breastfeeding initiation, intention and duration: A systematic review and dose-response meta-analysis
Source: Heliyon. 2020 Dec 7;6(12):e05622. doi: 10.1016/j.heliyon.2020.e05622 (PMC7725724; doi:10.1016/j.heliyon.2020.e05622)
Supplement: Appendix_2 [file mmc2.docx]

**The description of eligible studies reporting the breastfeeding initiation, intention and duration by pre-pregnancy BMI levels**

| **ID** | **First Author** | **Country** | **YOP** | **Study Design** | **Mean/**  **Median of Age** | **Week of pregnancy- BMI** | **Assessment of weight/height** | **BMI category**  **(kg/m^2^)** | **N_BMI group** | **BF & Initiation of BF (%)** | **Mean/Median of Duration BF**  **(week)** | **Intention of BF (%)** |
| --- | --- | --- | --- | --- | --- | --- | --- | --- | --- | --- | --- | --- |
| 1 | NJ Sebire [33] | United Kingdom | 2001 | retrospective | - | at booking | recorded | 20-24.9 | 176923 | 96.3 |  |  |
|  |  |  |  |  |  |  |  | 25–29.9 | 79014 | 96.5 |  |  |
|  |  |  |  |  |  |  |  | >30 | 31276 | 96.3 |  |  |
| 2 | Jennifer L Baker [63] | Denmark | 2004 | prospective study | 30.8±4 | pre-pregnancy | reported | <18.5 | 166 |  | 15.5±5.9**^*^** |  |
|  |  |  |  |  |  |  |  | 18.5-24.9 | 2611 |  | 16.3±4.9 |  |
|  |  |  |  |  |  |  |  | 25-29.9 | 717 |  | 15.6±5.1 |  |
|  |  |  |  |  |  |  |  | >30 | 274 |  | 14.9±5.5 |  |
|  |  |  |  |  |  |  |  | <18.5 | 166 |  | 29.5±16.4**^**^** |  |
|  |  |  |  |  |  |  |  | 18.5-24.9 | 2611 |  | 31.3±14.6 |  |
|  |  |  |  |  |  |  |  | 25-29.9 | 717 |  | 29.2±15 |  |
|  |  |  |  |  |  |  |  | >30 | 274 |  | 27.3±15.7 |  |
| 3 | Janet G. Kugyelka [34] | United States | 2004 | community-based study | 25±5.4  26.4±4.9  26.9±5.2  25.4±5.2  25.7±5.4  26±4.8  for each BMI category | pre-pregnancy | recorded | 19-26  (Black, within 2 h) | 305 | 75.1 |  |  |
|  |  |  |  |  |  |  |  | 26-29 | 95 | 69.2 |  |  |
|  |  |  |  |  |  |  |  | >29 | 240 | 63.8 |  |  |
|  |  |  |  |  |  |  |  | 19-26 (Hispanic) | 325 | 71.8 |  |  |
|  |  |  |  |  |  |  |  | 26-29 | 98 | 66.7 |  |  |
|  |  |  |  |  |  |  |  | >29 | 164 | 61.5 |  |  |
|  |  |  |  |  |  |  |  | 19-26  (Black, At least 1/5 feeds) | 305 | 94.9 |  |  |
|  |  |  |  |  |  |  |  | 26-29 | 95 | 91.5 |  |  |
|  |  |  |  |  |  |  |  | >29 | 240 | 93.1 |  |  |
|  |  |  |  |  |  |  |  | 19-26 (Hispanic) | 325 | 90.4 |  |  |
|  |  |  |  |  |  |  |  | 26-29 | 98 | 87.8 |  |  |
|  |  |  |  |  |  |  |  | >29 | 164 | 87.6 |  |  |
| 4 | Andrej M. Grjibovski [64] | Russia | 2005 | community-based cohort | - | pre-pregnancy | recorded | <18.5 | 59 | 98.3 | 5.5 (3-12) |  |
|  |  |  |  |  |  |  |  | 18.5-24.9 | 969 | 98.7 | 5 (3-9) |  |
|  |  |  |  |  |  |  |  | 25-29.9 | 50 | 100 | 4.25 (2-8) |  |
| 5 | Jane A. Scott [27] | Australia | 2006 | prospective cohort | - | pre-pregnancy | - | <25 | 362 | 95.6**^*^** |  |  |
|  |  |  |  |  |  |  |  | 25-29.9 | 106 | 91.5 |  |  |
|  |  |  |  |  |  |  |  | >30 | 86 | 90.7 |  |  |
|  |  |  |  |  |  |  |  | <25 (at 7 day) | 362 | 82.1 |  |  |
|  |  |  |  |  |  |  |  | 25-29.9 | 106 | 70.5 |  |  |
|  |  |  |  |  |  |  |  | >30 | 86 | 63.1 |  |  |
|  |  |  |  |  |  |  |  | <25 (at 1 mo) | 362 | 60.1 |  |  |
|  |  |  |  |  |  |  |  | 25-29.9 | 106 | 59.1 |  |  |
|  |  |  |  |  |  |  |  | >30 | 86 | 52.2 |  |  |
|  |  |  |  |  |  |  |  | <25 (at 3 mo) | 362 | 44.1 |  |  |
|  |  |  |  |  |  |  |  | 25-29.9 | 106 | 38.8 |  |  |
|  |  |  |  |  |  |  |  | >30 | 86 | 30.3 |  |  |
|  |  |  |  |  |  |  |  | <25 (at 6 mo) | 362 | 12.7 |  |  |
|  |  |  |  |  |  |  |  | 25-29.9 | 106 | 13.5 |  |  |
|  |  |  |  |  |  |  |  | >30 | 86 | 13 |  |  |
|  |  |  |  |  |  |  |  | <25 (at 7 day) | 362 | 91.8**^**^** |  |  |
|  |  |  |  |  |  |  |  | 25-29.9 | 106 | 89.3 |  |  |
|  |  |  |  |  |  |  |  | >30 | 86 | 85.9 |  |  |
|  |  |  |  |  |  |  |  | <25 (at 1 mo) | 362 | 80.1 |  |  |
|  |  |  |  |  |  |  |  | 25-29.9 | 106 | 82.4 |  |  |
|  |  |  |  |  |  |  |  | >30 | 86 | 72.4 |  |  |
|  |  |  |  |  |  |  |  | <25 (at 3 mo) | 362 | 65.2 |  |  |
|  |  |  |  |  |  |  |  | 25-29.9 | 106 | 65.2 |  |  |
|  |  |  |  |  |  |  |  | >30 | 86 | 49.4 |  |  |
|  |  |  |  |  |  |  |  | <25 (at 6 mo) | 362 | 49 |  |  |
|  |  |  |  |  |  |  |  | 25-29.9 | 106 | 49.3 |  |  |
|  |  |  |  |  |  |  |  | >30 | 86 | 35.7 |  |  |
|  |  |  |  |  |  |  |  | <25 (at 12 mo) | 362 | 20.7 |  |  |
|  |  |  |  |  |  |  |  | 25-29.9 | 106 | 18.7 |  |  |
|  |  |  |  |  |  |  |  | >30 | 86 | 15.6 |  |  |
| 6 | Wendy Hazel Oddy [65] | Australia | 2006 | prospective cohort study | - | pre-pregnancy | measured | 18.5-24.9 | 1479 | 91.8 |  |  |
|  |  |  |  |  |  |  |  | ≥25 | 324 | 87.3 |  |  |
|  |  |  |  |  |  |  |  | 18.5-24.9 (<2 mo) | 1479 | 70.8 |  |  |
|  |  |  |  |  |  |  |  | 25-29.9 | 211 | 15 |  |  |
|  |  |  |  |  |  |  |  | ≥30 | 113 | 9.9 |  |  |
|  |  |  |  |  |  |  |  | 18.5-24.9 (<4 mo) | 1479 | 76.6 |  |  |
|  |  |  |  |  |  |  |  | 25-29.9 | 211 | 14.5 |  |  |
|  |  |  |  |  |  |  |  | ≥30 | 113 | 8.9 |  |  |
|  |  |  |  |  |  |  |  | 18.5-24.9 (<6 mo) | 1479 | 78.6 |  |  |
|  |  |  |  |  |  |  |  | 25-29.9 | 211 | 13.6 |  |  |
|  |  |  |  |  |  |  |  | ≥30 | 113 | 7.7 |  |  |
| 7 | Julie A. Hilson [42] | United States | 2006 | - | 27.3±5.1  28.5±5.1  28±4.8  27.9±4.9  for each category | pre-pregnancy | self-reported | <19.8 | 364 |  | 1.7**^*^** |  |
|  |  |  |  |  |  |  |  | 19.8-26 | 1522 |  | 2 |  |
|  |  |  |  |  |  |  |  | 26.1-29 | 354 |  | 1.7 |  |
|  |  |  |  |  |  |  |  | >29 | 543 |  | 1.1 |  |
|  |  |  |  |  |  |  |  | <19.8 | 364 |  | 8**^**^** |  |
|  |  |  |  |  |  |  |  | 19.8-26 | 1522 |  | 8 |  |
|  |  |  |  |  |  |  |  | 26.1-29 | 354 |  | 7 |  |
|  |  |  |  |  |  |  |  | >29 | 543 |  | 2 |  |
| 8 | Jennifer L Baker [66] | Denmark | 2007 | cohort | 30.5±4.2 | pre-pregnancy | self-reported | <18.5 | 1569 | 98.2**^*^** | 14.9±6.3**^*^** |  |
|  |  |  |  |  |  |  |  | 18.5-24.9 | 25316 | 97.7 | 15.3±6 |  |
|  |  |  |  |  |  |  |  | 25-29.9 | 7447 | 96.3 | 14.2±6.6 |  |
|  |  |  |  |  |  |  |  | 30-34.9 | 2312 | 94.9 | 12.8±7 |  |
|  |  |  |  |  |  |  |  | 35-39.9 | 600 | 92.3 | 12.8±7 |  |
|  |  |  |  |  |  |  |  | ≥40 | 215 | 94 | 11.2±7.4 |  |
|  |  |  |  |  |  |  |  | <18.5 | 1569 | 64**^**^** | 13.3±7.4**^**^** |  |
|  |  |  |  |  |  |  |  | 18.5-24.9 | 25316 | 63.7 | 14.1±7.5 |  |
|  |  |  |  |  |  |  |  | 25-29.9 | 7447 | 67.3 | 12.9±7.4 |  |
|  |  |  |  |  |  |  |  | 30-34.9 | 2312 | 72.4 | 11.5±7.4 |  |
|  |  |  |  |  |  |  |  | 35-39.9 | 600 | 71.7 | 11.5±7.4 |  |
|  |  |  |  |  |  |  |  | ≥40 | 215 | 73.5 | 9.6±6.7 |  |
| 9 | Susan M. Donath [67] | Australia | 2008 | Longitudinal Study | 30.41 | pre-pregnancy | self-reported | 20-24.9 | 1567 | 95.1 |  |  |
|  |  |  |  |  |  |  |  | 25-30 | 890 | 92.8 |  |  |
|  |  |  |  |  |  |  |  | ≥30 | 618 | 87.1 |  |  |
|  |  |  |  |  |  |  |  | 20-24.9 (at first week) | 1567 | 91.7 |  |  |
|  |  |  |  |  |  |  |  | 25-30 | 890 | 87.1 |  |  |
|  |  |  |  |  |  |  |  | ≥30 | 618 | 77.7 |  |  |
|  |  |  |  |  |  |  |  | 20-24.9 (at 6 mo) | 1567 | 63.8 |  |  |
|  |  |  |  |  |  |  |  | 25-30 | 890 | 52.6 |  |  |
|  |  |  |  |  |  |  |  | ≥30 | 618 | 43.9 |  |  |
| 10 | Elise Mok [28] | France | 2008 | case-control study | - | pre-pregnancy | self-reported | 18.5-25 (at 1 mo) | 863 | 73.3**^*^** |  |  |
|  |  |  |  |  |  |  |  | ≥30 | 141 | 50 |  |  |
|  |  |  |  |  |  |  |  | 18.5-25 (at 3 mo) | 863 | 46.6 |  |  |
|  |  |  |  |  |  |  |  | ≥30 | 141 | 21.6 |  |  |
|  |  |  |  |  |  |  |  | 18.5-25 (at 1 mo) | 863 | 83.3**^**^** |  |  |
|  |  |  |  |  |  |  |  | ≥30 | 141 | 71.6 |  |  |
|  |  |  |  |  |  |  |  | 18.5-25 (at 3 mo) | 863 | 65 |  |  |
|  |  |  |  |  |  |  |  | ≥30 | 141 | 51.6 |  |  |
| 11 | Panagiota Kitsantas [68] | United States | 2010 | longitudinal study-birth cohort | - | pre-pregnancy | self-reported | <19.8 (medical conditions) |  | 54.2 |  |  |
|  |  |  |  |  |  |  |  | 19.8-26 |  | 11.4 |  |  |
|  |  |  |  |  |  |  |  | 26-29 |  | 15.4 |  |  |
|  |  |  |  |  |  |  |  | >29 |  | 18.9 |  |  |
|  |  |  |  |  |  |  |  | <19.8 (no medical condition) |  | 57.9 |  |  |
|  |  |  |  |  |  |  |  | 19.8-26 |  | 15.8 |  |  |
|  |  |  |  |  |  |  |  | 26-29 |  | 12.1 |  |  |
|  |  |  |  |  |  |  |  | >29 |  | 14.2 |  |  |
| 12 | Liu J [35] | United States | 2010 | population-based surveillance | - | pre-pregnancy | self-reported | <18.5 (White) | 177 |  | 10 mo |  |
|  |  |  |  |  |  |  |  | 18.5-24.9 | 1322 |  | 14 |  |
|  |  |  |  |  |  |  |  | 25-29.9 | 513 |  | 12.9 |  |
|  |  |  |  |  |  |  |  | 30-34.9 | 265 |  | 9.8 |  |
|  |  |  |  |  |  |  |  | ≥35 | 179 |  | 5.7 |  |
|  |  |  |  |  |  |  |  | <18.5 (Black) | 79 |  | 3.7 |  |
|  |  |  |  |  |  |  |  | 18.5-24.9 | 533 |  | 7.9 |  |
|  |  |  |  |  |  |  |  | 25-29.9 | 384 |  | 11 |  |
|  |  |  |  |  |  |  |  | 30-34.9 | 240 |  | 8.1 |  |
|  |  |  |  |  |  |  |  | ≥35 | 210 |  | 8.5 |  |
| 13 | Stephanie A Leonard [29] | United States | 2011 | longitudinal study | - | pre-pregnancy | self-reported | 18.5-24.9 | 1144 | 82.2 | - |  |
|  |  |  |  |  |  |  |  | 25-29.9 | 607 | 81.9 | 30.1 |  |
|  |  |  |  |  |  |  |  | ≥30 | 537 | 83.1 | 25.8 |  |
|  |  |  |  |  |  |  |  | 18.5-24.9 (2 mo) | 1144 | 85.2 |  |  |
|  |  |  |  |  |  |  |  | 25-29.9 | 607 | 84.8 |  |  |
|  |  |  |  |  |  |  |  | ≥30 | 537 | 89.6 |  |  |
|  |  |  |  |  |  |  |  | 18.5-24.9 (5 mo) | 1144 | 76.2 |  |  |
|  |  |  |  |  |  |  |  | 25-29.9 | 607 | 82.8 |  |  |
|  |  |  |  |  |  |  |  | ≥30 | 537 | 78.4 |  |  |
| 14 | Katrina M. Krause [22] | United States | 2011 | secondary analysis | - | pre-pregnancy | self-reported | 25-29.9 | 180 | 75 |  |  |
|  |  |  |  |  |  |  |  | 30-34.9 | 141 | 72.3 |  |  |
|  |  |  |  |  |  |  |  | 35-39.9 | 70 | 60 |  |  |
|  |  |  |  |  |  |  |  | 40 | 59 | 52.5 |  |  |
| 15 | Isabelle Guelinckx [30] | Belgium | 2012 | retrospective study | 29 | pre-pregnancy | self-reported | <18.5 | 50 | 62 | 3 mo | 64 |
|  |  |  |  |  |  |  |  | 18.5-24.9 | 50 | 92 | 3 | 92 |
|  |  |  |  |  |  |  |  | 25-29.9 | 50 | 80 | 3 | 80 |
|  |  |  |  |  |  |  |  | ≥30 | 50 | 68 | 1.8 | 68 |
|  |  |  |  |  |  |  |  | <18.5 (1 mo) | 50 | 52**^*^** |  |  |
|  |  |  |  |  |  |  |  | 18.5-24.9 | 50 | 70 |  |  |
|  |  |  |  |  |  |  |  | 25-29.9 | 50 | 56 |  |  |
|  |  |  |  |  |  |  |  | ≥30 | 50 | 34 |  |  |
|  |  |  |  |  |  |  |  | <18.5 (3 mo) | 50 | 36 |  |  |
|  |  |  |  |  |  |  |  | 18.5-24.9 | 50 | 60 |  |  |
|  |  |  |  |  |  |  |  | 25-29.9 | 50 | 40 |  |  |
|  |  |  |  |  |  |  |  | ≥30 | 50 | 22 |  |  |
| 16 | Siv Tone Natland [69] | Norway | 2012 | population-based prospective study | 29.1 ±3.9 | pre-pregnancy | recorded | <25 | 314 |  | 7 mo |  |
|  |  |  |  |  |  |  |  | ≥25 | 30 |  | 4.5 |  |
| 17 | Lindsay A. Thompson [36] | United States | 2013 | - | - | pre-pregnancy | - | <18.5 | 62745 | 74.2 |  |  |
|  |  |  |  |  |  |  |  | 18.5-24.9 | 592593 | 80.4 |  |  |
|  |  |  |  |  |  |  |  | 25-29.9 | 267248 | 49.1 |  |  |
|  |  |  |  |  |  |  |  | ≥30 | 220770 | 74 |  |  |
| 18 | Laura E Hauff [37] | United States | 2014 | longitudinal cohort study | - | pre-pregnancy | self-reported | <25 | 1406 |  |  | 87.2 |
|  |  |  |  |  |  |  |  | 25-29.9 | 741 |  |  | 87.2 |
|  |  |  |  |  |  |  |  | ≥30 | 677 |  |  | 83.9 |
|  |  |  |  |  |  |  |  | <25 | 1406 | 86.4 |  |  |
|  |  |  |  |  |  |  |  | 25-29.9 | 741 | 87.3 |  |  |
|  |  |  |  |  |  |  |  | ≥30 | 677 | 82.9 |  |  |
|  |  |  |  |  |  |  |  | <25 | 1406 |  | 14.7**^*^** |  |
|  |  |  |  |  |  |  |  | 25-29.9 | 741 |  | 14.1 |  |
|  |  |  |  |  |  |  |  | ≥30 | 677 |  | 8.1 |  |
|  |  |  |  |  |  |  |  | <25 | 1406 |  | 34.4**^**^** |  |
|  |  |  |  |  |  |  |  | 25-29.9 | 741 |  | 30.1 |  |
|  |  |  |  |  |  |  |  | ≥30 | 677 |  | 25.8 |  |
| 19 | Saba W. Masho [70] | United States | 2015 | national surveillance | - | pre-pregnancy | self-reported | <18.5 | 213108 | 77.9 |  |  |
|  |  |  |  |  |  |  |  | 18.5-24.9 | 2477498 | 83.3 |  |  |
|  |  |  |  |  |  |  |  | 25-29.9 | 1186841 | 81.4 |  |  |
|  |  |  |  |  |  |  |  | ≥30 | 1041886 | 77.5 |  |  |
| 20 | Ruth Lucas [31] | United States | 2015 | Longitudinal descriptive | 31 ±5.41 | pre-pregnancy | telephone call | <24.9 (hoapital) | 17 | 23.5**^*^** |  |  |
|  |  |  |  |  |  |  |  | 25-29.9 | 40 | 25 |  |  |
|  |  |  |  |  |  |  |  | 30.34.9 | 25 | 36 |  |  |
|  |  |  |  |  |  |  |  | 35-39.9 | 13 | 38.5 |  |  |
|  |  |  |  |  |  |  |  | >40 | 21 | 9.5 |  |  |
|  |  |  |  |  |  |  |  | <24.9 (week 4) | 17 | 11.8 |  |  |
|  |  |  |  |  |  |  |  | 25-29.9 | 40 | 25 |  |  |
|  |  |  |  |  |  |  |  | 30.34.9 | 25 | 12 |  |  |
|  |  |  |  |  |  |  |  | 35-39.9 | 13 | 30.8 |  |  |
|  |  |  |  |  |  |  |  | >40 | 21 | 38.1 |  |  |
| 21 | Julie Verret-Chalifour [71] | Canada | 2015 | prospective cohort study | - | pre-pregnancy | self-reported | <18.5 | 337 | 85.5 |  |  |
|  |  |  |  |  |  |  |  | 18.5-24.9 | 4105 | 88 |  |  |
|  |  |  |  |  |  |  |  | 25-29.9 | 1317 | 86.3 |  |  |
|  |  |  |  |  |  |  |  | ≥30 | 833 | 79.9 |  |  |
| 22 | Anna Winkvist [11] | Norway | 2015 | a prospective, population-based pregnancy cohort | - | pre-pregnancy | self-reported | <18.5 | 1490 | 99 |  |  |
|  |  |  |  |  |  |  |  | 18.5-24.9 | 32732 | 99.6 |  |  |
|  |  |  |  |  |  |  |  | 25-29.9 | 10828 | 98.8 |  |  |
|  |  |  |  |  |  |  |  | 30-34.9 | 3377 | 98.7 |  |  |
|  |  |  |  |  |  |  |  | 35-39.9 | 1192 | 97.4 |  |  |
|  |  |  |  |  |  |  |  | <18.5 (for 4 mo) | 1490 | 57.8**^*^** |  |  |
|  |  |  |  |  |  |  |  | 18.5-24.9 | 32732 | 62.9 |  |  |
|  |  |  |  |  |  |  |  | 25-29.9 | 10828 | 54.4 |  |  |
|  |  |  |  |  |  |  |  | 30-34.9 | 3377 | 45.4 |  |  |
|  |  |  |  |  |  |  |  | 35-39.9 | 1192 | 37.8 |  |  |
|  |  |  |  |  |  |  |  | <18.5 (for 6 mo) | 1490 | 14.2 |  |  |
|  |  |  |  |  |  |  |  | 18.5-24.9 | 32732 | 14.8 |  |  |
|  |  |  |  |  |  |  |  | 25-29.9 | 10828 | 11.6 |  |  |
|  |  |  |  |  |  |  |  | 30-34.9 | 3377 | 10.3 |  |  |
|  |  |  |  |  |  |  |  | 35-39.9 | 1192 | 8.9 |  |  |
|  |  |  |  |  |  |  |  | <18.5 (for 4 mo) | 1490 | 85.1**^**^** |  |  |
|  |  |  |  |  |  |  |  | 18.5-24.9 | 32732 | 90.6 |  |  |
|  |  |  |  |  |  |  |  | 25-29.9 | 10828 | 85.7 |  |  |
|  |  |  |  |  |  |  |  | 30-34.9 | 3377 | 77.9 |  |  |
|  |  |  |  |  |  |  |  | 35-39.9 | 1192 | 71.3 |  |  |
|  |  |  |  |  |  |  |  | <18.5 (for 6 mo) | 1490 | 77 |  |  |
|  |  |  |  |  |  |  |  | 18.5-24.9 | 32732 | 83.8 |  |  |
|  |  |  |  |  |  |  |  | 25-29.9 | 10828 | 77.4 |  |  |
|  |  |  |  |  |  |  |  | 30-34.9 | 3377 | 68.4 |  |  |
|  |  |  |  |  |  |  |  | 35-39.9 | 1192 | 58.7 |  |  |
| 23 | Kylee Cox [32] | Australia | 2015 | - | 30.2±5.2 | pre-pregnancy | - | <30 | 290 | 84.8**^*^** | 22**^*^** |  |
|  |  |  |  |  |  |  |  | ≥30 | 47 | 73.4 | 40 |  |
|  |  |  |  |  |  |  |  | <30 (for 4 mo) | 290 | 39.4 |  |  |
|  |  |  |  |  |  |  |  | ≥30 | 47 | 23.2 |  |  |
|  |  |  |  |  |  |  |  | <30 (for 6 mo) | 290 | 7.1 |  |  |
|  |  |  |  |  |  |  |  | ≥30 | 47 | 0 |  |  |
|  |  |  |  |  |  |  |  | <30 | 342 | 97.1**^**^** |  |  |
|  |  |  |  |  |  |  |  | ≥30 | 64 | 94.2 |  |  |
|  |  |  |  |  |  |  |  | <30 (for 4 mo) | 342 | 79.2 |  |  |
|  |  |  |  |  |  |  |  | ≥30 | 64 | 68.9 |  |  |
|  |  |  |  |  |  |  |  | <30 (for 6 mo) | 342 | 72.3 |  |  |
|  |  |  |  |  |  |  |  | ≥30 | 64 | 60.4 |  |  |
|  |  |  |  |  |  |  |  | <30 (for 12 mo) | 342 | 39.1 |  |  |
|  |  |  |  |  |  |  |  | ≥30 | 64 | 35.2 |  |  |
| 24 | Josefa L. Martinez [23] | United States | 2016 | - | 25.6±5.5 | pre-pregnancy | self-reported or measured | 25-29.9 | 238 | 28.6**^*^** |  |  |
|  |  |  |  |  |  |  |  | 30-34.9 | 132 | 18.2 |  |  |
|  |  |  |  |  |  |  |  | 35-39.9 | 64 | 10.9 |  |  |
|  |  |  |  |  |  |  |  | ≥40 | 46 | 13 |  |  |
| 25 | Contact H Castillo [72] | Brazil | 2016 | prospective cohort | 26.1 | pre-pregnancy | extracted from prenatal card or when absent by self-report | <18.5 | 181 | 21**^*^** | 1 mo**^*^** |  |
|  |  |  |  |  |  |  |  | 18.5-24.9 | 2286 | 28.6 | 1.6 |  |
|  |  |  |  |  |  |  |  | 25-29.9 | 877 | 27.7 | 1.5 |  |
|  |  |  |  |  |  |  |  | ≥30 | 413 | 25.5 | 1.2 |  |
|  |  |  |  |  |  |  |  | <18.5 | 181 |  | 5 mo |  |
|  |  |  |  |  |  |  |  | 18.5-24.9 | 2286 |  | 7 |  |
|  |  |  |  |  |  |  |  | 25-29.9 | 877 |  | 7 |  |
|  |  |  |  |  |  |  |  | ≥30 | 413 |  | 6 |  |
| 26 | Susan J.de Jersey [7] | Australia | 2017 | prospective, observational study | 30±5 | pre-pregnancy | self-reported | <25 | 267 | 86**^*^** |  | 62 |
|  |  |  |  |  |  |  |  | ≥25 | 135 | 73 |  | 53 |
|  |  |  |  |  |  |  |  | <25 (at 4 mo) | 267 | 66 |  |  |
|  |  |  |  |  |  |  |  | ≥25 | 135 | 67 |  |  |
| 27 | Naila Ramji [13] | Canada | 2017 | population-based retrospective cohort | - | pre-pregnancy | recorded | 18.5-24.9 | 5685 | 71.7 |  |  |
|  |  |  |  |  |  |  |  | 25-29.9 | 3394 | 69.1 |  |  |
|  |  |  |  |  |  |  |  | 30-39.9 | 3394 | 61.6 |  |  |
|  |  |  |  |  |  |  |  | 40-49.9 | 535 | 54.2 |  |  |
|  |  |  |  |  |  |  |  | ≥50 | 71 | 42.3 |  |  |
| 28 | Xing-Yong Tao [73] | China | 2017 | cohort | 26·4±3·6 | first prenatal visit | weight was self-reported/ height was measured | <18.5 | 605 | 95 | 7 mo**^**^** |  |
|  |  |  |  |  |  |  |  | 18.5-23.9 | 2209 | 96.4 | 7 |  |
|  |  |  |  |  |  |  |  | 24-27.9 | 300 | 94.7 | 6 |  |
|  |  |  |  |  |  |  |  | ≥28 | 82 | 93.9 | 3 |  |
| 29 | Afsaneh Tehrania [2] | Iran | 2017 | prospective cohort | 26.68 | In the first prenatal visit | self-report | <25 | 358 | 90.8 |  | 89.4 |
|  |  |  |  |  |  |  |  | ≥25 | 235 | 86.8 |  | 84.2 |
|  |  |  |  |  |  |  |  | <25 (for 6 mo) | 358 | 82.7 |  |  |
|  |  |  |  |  |  |  |  | ≥25 | 235 | 80.9 |  |  |
| 30 | Julie Boudet‐Berquier [74] | France | 2018 | prospective birth cohort | - | pre-pregnancy | self‐reported | <18.5 | 161 | 77**^*^** | 30 day**^*^** |  |
|  |  |  |  |  |  |  |  | 18.5-24.9 | 1521 | 83.43 | 30 |  |
|  |  |  |  |  |  |  |  | 25-29.9 | 426 | 78.4 | 34 |  |
|  |  |  |  |  |  |  |  | ≥30 | 208 | 67.8 | 17 |  |
| 31 | Tanara Vogel Pinheiro [75] | Brazil | 2018 | prospective follow-up | 26.71 | pre-pregnancy | the prenatal care booklet | 18.5-24.9 | 85 | 100 |  |  |
|  |  |  |  |  |  |  |  | ≥25 | 64 | 95.2 |  |  |
| 32 | Nicole E Marshall [41] | United States | 2018 | cohort longitudinal study | 32.8±4.6  34.5±4.8  30.4±7.8  for each category | pre-pregnancy | self-reported | 18.5-24.9  (at 6 weeks) | 64 | 90.6**^*^** |  |  |
|  |  |  |  |  |  |  |  | ≥25 | 76 | 67.1 |  |  |
|  |  |  |  |  |  |  |  | 18.5-24.9  (at 6 mo) | 64 | 79 |  |  |
|  |  |  |  |  |  |  |  | ≥25 | 76 | 36.7 |  |  |
|  |  |  |  |  |  |  |  | 18.5-24.9 | 64 |  | 15.2±4.9 mo |  |
|  |  |  |  |  |  |  |  | 25-29.9 | 50 |  | 13.8±9.2 |  |
|  |  |  |  |  |  |  |  | ≥30 | 26 |  | 11.8±4.4 |  |
| 33 | Tammy Campbell [40] | United Kingdom | 2018 | cohort study | - | pre-pregnancy | self-reported | <18.5 | 999 | 80.2 |  |  |
|  |  |  |  |  |  |  |  | 18.5-24.9 | 11266 | 84.3 |  |  |
|  |  |  |  |  |  |  |  | 25-29.9 | 3373 | 77.1 |  |  |
|  |  |  |  |  |  |  |  | ≥30 | 1475 | 76.7 |  |  |

**The description of eligible studies reporting the effect of pre-pregnancy BMI levels on the breastfeeding initiation, intention and duration**

| **ID** | **First Author** | **Country** | **YOP** | **Study Design** | **Mean/**  **Median of Age** | **Assessment of weight/ height** | **BMI category**  **(kg/m^2^)** | **N_BMI group** | **Crude OR**  **(95% CI)** | **Adjusted OR**  **(95% CI)** | **Crude RR**  **(95% CI)** | **Adjusted RR**  **(95% CI)** | **Crude HR**  **(95% CI)** | **Adjusted HR**  **(95% CI)** |
| --- | --- | --- | --- | --- | --- | --- | --- | --- | --- | --- | --- | --- | --- | --- |
| 1 | Ingrid H E Rutishauser [76] | Australia | 1992 | prospective cohor | 25.3±4 | measured | ≤26 |  |  |  |  | 1^*^ |  |  |
|  |  |  |  |  |  |  | ≥26 |  |  |  |  | 1.5  (1.11-2.04) |  |  |
| 2 | NJ Sebire [33] | United Kingdom | 2001 | retrospective | - | recorded | 20-24.9 | 176923 | 1 | 1^$^ |  |  |  |  |
|  |  |  |  |  |  |  | 25–29.9 | 79014 | 1.06  (1.01-1.11) | 0.86  (0.84-0.88) |  |  |  |  |
|  |  |  |  |  |  |  | >30 | 31276 | 1  (0.94-1.06) | 0.58  (0.56-0.6) |  |  |  |  |
| 3 | Janet G. Kugyelka [34] | United States | 2004 | community-based study | 25±5.4  26.4±4.9  26.9±5.2  25.4±5.2  25.7±5.4  26±4.8  for each BMI category | recorded | 19-26  (Black,  within 2 h) | 305 | 1^$$^ | 1 | 1^##^ | 1 |  |  |
|  |  |  |  |  |  |  | 26-29 | 95 | 1.74  (0.96-3.16) | 1.54  (0.83-2.83) | 0.88  (0.62-1.27) | 0.91  (0.62-1.17) |  |  |
|  |  |  |  |  |  |  | >29 | 240 | 1.28  (0.82-2) | 1.13  (0.71-1.82) | 0.83  (0.64-1.09) | 0.88  (0.66-1.17) |  |  |
|  |  |  |  |  |  |  | 19-26  (Hispanic) | 325 | 1 | 1 | 1 | 1 |  |  |
|  |  |  |  |  |  |  | 26-29 | 98 | 1.46  (0.84-2.55) | 1.3  (0.73-2.32) | 0.96  (0.67-1.38) | 0.93  (0.63-1.37) |  |  |
|  |  |  |  |  |  |  | >29 | 164 | 2.22  (1.42-3.84) | 1.92  (1.2-3.08) | 1.36  (1.1-1.84) | 1.45  (1.06-1.99) |  |  |
|  |  |  |  |  |  |  | 19-26  (Black, At least  1/5 feeds) | 305 |  |  | 1^###^ | 1 |  |  |
|  |  |  |  |  |  |  | 26-29 | 95 |  |  | 0.87  (0.6-1.26) | 0.88  (0.6-1.28) |  |  |
|  |  |  |  |  |  |  | >29 | 240 |  |  | 0.96  (0.73-1.26) | 1.04  (0.77-1.4) |  |  |
|  |  |  |  |  |  |  | 19-26 (Hispanic) | 325 |  |  | 1 | 1 |  |  |
|  |  |  |  |  |  |  | 26-29 | 98 |  |  | 1.14  (0.8-1.64) | 1.17  (0.8-1.72) |  |  |
|  |  |  |  |  |  |  | >29 | 164 |  |  | 1.41  (1.04-1.91) | 1.55  (1.13-2.13) |  |  |
| 4 | Andrej M. Grjibovski [64] | Russia | 2005 | community-based cohort | - | recorded | <18.5 | 59 | 0.85  (0.62-1.16) | 0.84  (0.62-1.16) |  |  |  |  |
|  |  |  |  |  |  |  | 18.5-24.9 | 969 | 1^#^ | 1 |  |  |  |  |
|  |  |  |  |  |  |  | 25-29.9 | 50 | 1.22  (0.9-1.65) | 1.27  (0.93-1.73) |  |  |  |  |
| 5 | Jane A. Scott [27] | Australia | 2006 | prospective cohort | - | - | <25 | 362 | 1^**^ | 1 |  |  |  |  |
|  |  |  |  |  |  |  | 25-29.9 | 106 | 0.5  (0.21-1.16) | 0.5  (0.28-0.89) |  |  |  |  |
|  |  |  |  |  |  |  | >30 | 86 | 0.45  (0.19-1.09) | 0.63  (0.33-1.2) |  |  |  |  |
| 6 | Wendy Hazel Oddy [65] | Australia | 2006 | prospective cohort study | - | measured | 18.5-24.9 | 1479 |  |  |  |  |  | 1^***^ |
|  |  |  |  |  |  |  | ≥25 | 324 |  |  |  |  |  | 1.18  (1.05-1.35) |
|  |  |  |  |  |  |  | 18.5-24.9  (<2 mo) | 1479 | 1^***^ | 1 |  |  |  |  |
|  |  |  |  |  |  |  | 25-29.9 | 211 | 1.37  (1.06-1.78) | 1.52  (1.11-2.09) |  |  |  |  |
|  |  |  |  |  |  |  | ≥30 | 113 | 1.98  (1.41-2.78) | 2.08  (1.39-3.12) |  |  |  |  |
|  |  |  |  |  |  |  | 18.5-24.9  (<4 mo) | 1479 | 1 | 1 |  |  |  |  |
|  |  |  |  |  |  |  | 25-29.9 | 211 | 1.37  (1.07-1.75) | 1.62  (1.2-2.18) |  |  |  |  |
|  |  |  |  |  |  |  | ≥30 | 113 | 1.97  (1.4-2.77) | 1.98  (1.32-2.95) |  |  |  |  |
|  |  |  |  |  |  |  | 18.5-24.9  (<6 mo) | 1479 | 1 | 1 |  |  |  |  |
|  |  |  |  |  |  |  | 25-29.9 | 211 | 1.31  (1.02-1.69) | 1.53  (1.13-2.07) |  |  |  |  |
|  |  |  |  |  |  |  | ≥30 | 113 | 1.68  (1.18-2.38) | 1.54  (1.02-2.32) |  |  |  |  |
| 7 | Julie A. Hilson [42] | United States | 2006 | - | 27.3±5.1  28.5±5.1  28±4.8  27.9±4.9  for each category | self-reported | <19.8 (<IOM) | 88 |  | 1.21^$$^  (0.53-2.76) |  |  |  | 1.31^**^  (0.99-1.71) |
|  |  |  |  |  |  |  | 19.8-26 | 256 |  | 1.69  (0.99-2.88) |  |  |  | 1.19  (0.98-1.44) |
|  |  |  |  |  |  |  | 26.1-29 | 19 |  | 2.96  (0.9-9.79) |  |  |  | 1.59  (0.94-2.68) |
|  |  |  |  |  |  |  | >29 | 83 |  | 1.81  (0.86-3.83) |  |  |  | 1.37  (1.01-1.84) |
|  |  |  |  |  |  |  | <19.8  (within IOM) | 144 |  | 1.59  (0.82-3.07) |  |  |  | 1.04  (0.82-1.31) |
|  |  |  |  |  |  |  | 19.8-26 | 469 |  | 1 |  |  |  | 1 |
|  |  |  |  |  |  |  | 26.1-29 | 68 |  | 1.47  (0.61-3.53) |  |  |  | 1.13  (0.81-1.58) |
|  |  |  |  |  |  |  | >29 | 72 |  | 1.84  (0.83-4.11) |  |  |  | 1.5  (1.11-2.03) |
|  |  |  |  |  |  |  | <19.8 (>IOM) | 84 |  | 1.88  (0.91-3.92) |  |  |  | 1.39  (1.01-1.92) |
|  |  |  |  |  |  |  | 19.8-26 | 579 |  | 1.66  (1.05-2.63) |  |  |  | 1.14  (0.96-1.33) |
|  |  |  |  |  |  |  | 26.1-29 | 305 |  | 1.62  (0.9-2.91) |  |  |  | 1.27  (1.03-1.56) |
|  |  |  |  |  |  |  | >29 | 299 |  | 2.89  (1.78-4.69) |  |  |  | 1.78  (1.48-2.14) |
|  |  |  |  |  |  |  | <19.8 (<IOM) | 88 |  |  |  |  |  | 1.21^***^  (0.89-1.64) |
|  |  |  |  |  |  |  | 19.8-26 | 256 |  |  |  |  |  | 1.09  (0.88-1.35) |
|  |  |  |  |  |  |  | 26.1-29 | 19 |  |  |  |  |  | 0.84  (0.43-1.64) |
|  |  |  |  |  |  |  | >29 | 83 |  |  |  |  |  | 1.33  (0.96-1.85) |
|  |  |  |  |  |  |  | <19.8  (within IOM) | 144 |  |  |  |  |  | 0.95  (0.73-1.24) |
|  |  |  |  |  |  |  | 19.8-26 | 469 |  |  |  |  |  | 1 |
|  |  |  |  |  |  |  | 26.1-29 | 68 |  |  |  |  |  | 1.16  (0.82-1.64) |
|  |  |  |  |  |  |  | >29 | 72 |  |  |  |  |  | 1.57  (1.14-2.18) |
|  |  |  |  |  |  |  | <19.8 (>IOM) | 84 |  |  |  |  |  | 1.34  (0.95-1.88) |
|  |  |  |  |  |  |  | 19.8-26 | 579 |  |  |  |  |  | 1.09  (0.92-1.88) |
|  |  |  |  |  |  |  | 26.1-29 | 305 |  |  |  |  |  | 1.24  (0.98-1.55) |
|  |  |  |  |  |  |  | >29 | 299 |  |  |  |  |  | 1.99  (1.64-2.43) |
| 8 | Jennifer L Baker [66] | Denmark | 2007 | cohort | 30.5±4.2 | self-reported | <18.5 | 1569 |  |  |  | 0.97  (0.92-1.02) |  |  |
|  |  |  |  |  |  |  | 18.5-24.9 | 25316 |  |  |  | 1^##^ |  |  |
|  |  |  |  |  |  |  | 25-29.9 | 7447 |  |  |  | 1.07  (1.04-1.1) |  |  |
|  |  |  |  |  |  |  | 30-34.9 | 2312 |  |  |  | 1.19  (1.13-1.24) |  |  |
|  |  |  |  |  |  |  | 35-39.9 | 600 |  |  |  | 1.2  (1.1-1.31) |  |  |
|  |  |  |  |  |  |  | ≥40 | 215 |  |  |  | 1.4  (1.21-1.63) |  |  |
|  |  |  |  |  |  |  | <18.5 | 1569 |  |  |  | 0.94  (0.89-1) |  |  |
|  |  |  |  |  |  |  | 18.5-24.9 | 25316 |  |  |  | 1^###^ |  |  |
|  |  |  |  |  |  |  | 25-29.9 | 7447 |  |  |  | 1.12  (1.09-1.16) |  |  |
|  |  |  |  |  |  |  | 30-34.9 | 2312 |  |  |  | 1.24  (1.18-1.31) |  |  |
|  |  |  |  |  |  |  | 35-39.9 | 600 |  |  |  | 1.37  (1.25-1.5) |  |  |
|  |  |  |  |  |  |  | ≥40 | 215 |  |  |  | 1.39  (1.19-1.63) |  |  |
| 9 | Susan M. Donath [67] | Australia | 2008 | longitudinal study | 30.41 | self-reported | 20-24.9 | 1567 |  | 1^$$^ |  |  |  |  |
|  |  |  |  |  |  |  | 25-30 | 890 |  | 1.3  (0.91-1.84) |  |  |  |  |
|  |  |  |  |  |  |  | >30 | 618 |  | 2.1  (1.49-2.96) |  |  |  |  |
|  |  |  |  |  |  |  | 20-24.9  (at first week) | 1567 |  | 1^*^ |  |  |  |  |
|  |  |  |  |  |  |  | 25-30 | 890 |  | 1.52  (1.02-2.28) |  |  |  |  |
|  |  |  |  |  |  |  | >30 | 618 |  | 2.54  (1.7-3.79) |  |  |  |  |
|  |  |  |  |  |  |  | 20-24.9  (at 6 mo) | 1567 |  | 1 |  |  |  |  |
|  |  |  |  |  |  |  | 25-30 | 890 |  | 1.26  (1.04-1.53) |  |  |  |  |
|  |  |  |  |  |  |  | >30 | 618 |  | 1.38  (1.1-1.73) |  |  |  |  |
|  |  |  |  |  |  |  | 20-24.9  (at 6 mo) | 1567 |  | 1^$$^ |  |  |  |  |
|  |  |  |  |  |  |  | 25-30 | 890 |  | 1.34  (1.12-1.6) |  |  |  |  |
|  |  |  |  |  |  |  | >30 | 618 |  | 1.68  (1.37-2.06) |  |  |  |  |
| 10 | Yannis Manios [77] | Greece | 2008 | cross-sectional | - | self-reported | <19.8 | 368 | 0.79  (0.63-0.98) | 0.84  (0.66-1.07) |  |  |  |  |
|  |  |  |  |  |  |  | 19.8-26 | 1272 | 1^$$^ | 1 |  |  |  |  |
|  |  |  |  |  |  |  | 26-29 | 150 | 1.28  (0.93-1.78) | 1.3  (0.91-1.84) |  |  |  |  |
|  |  |  |  |  |  |  | >29 | 104 | 2.93  (1.87-4.57) | 2.86  (1.74-4.7) |  |  |  |  |
| 11 | Heather L. Kehler [78] | Canada | 2009 | cohort | - | self-reported | <18.5 | 32 | 1.22  (0.55-2.71) | 1 |  |  |  |  |
|  |  |  |  |  |  |  | 18.5-24.9 | 468 | 1^*^ |  |  |  |  |  |
|  |  |  |  |  |  |  | 25-29.9 | 168 | 1.47  (1-2.18) |  |  |  |  |  |
|  |  |  |  |  |  |  | ≥30 | 85 | 2.39  (1.48-3.88) | 2.13  (1.29-3.53) |  |  |  |  |
| 12 | Lenie van Rossem [1] | Netherlands | 2009 | population-based, prospective cohort study | 31.7±4.22 | self-reported | <25 | 1964 |  | 1^$^ |  |  |  |  |
|  |  |  |  |  |  |  | 25-30 | 411 |  | 0.91  (0.64-1.29) |  |  |  |  |
|  |  |  |  |  |  |  | ≥30 | 158 |  | 0.5  (0.33-0.77) |  |  |  |  |
| 13 | Panagiota Kitsantas [68] | United States | 2010 | longitudinal study-birth cohort | - | self-reported | <19.8 (medical conditions) |  | 1.4  (1.08-1.81) | 1.21  (0.91-1.59) |  |  |  | 0.96  (0.87-1.06) |
|  |  |  |  |  |  |  | 19.8-26 |  | 1^$$^ | 1 |  |  |  | 1^#^ |
|  |  |  |  |  |  |  | 26-29 |  | 0.97  (0.75-1.24) | 1.04  (0.91-1.59) |  |  |  | 1.05  (0.97-1.13) |
|  |  |  |  |  |  |  | >29 |  | 1.47  (1.18-1.81) | 1.37  (1.09-1.72) |  |  |  |  |
|  |  |  |  |  |  |  | <19.8  (no medical conditions) |  | 1.09  (0.86-1.38) | 0.96  (0.75-1.24) |  |  |  | 1.08  (0.98-1.19) |
|  |  |  |  |  |  |  | 19.8-26 |  | 1^$$^ | 1 |  |  |  | 1^#^ |
|  |  |  |  |  |  |  | 26-29 |  | 1.11  (0.85-1.44) | 1.1  (0.78-1.36) |  |  |  | 1.11  (1.1-1.21) |
|  |  |  |  |  |  |  | >29 |  | 1.52  (1.21-1.91) | 1.18  (0.92-1.51) |  |  |  |  |
| 14 | Liu J [35] | United States | 2010 | population-based surveillance | - | self-reported | <18.5  (White) | 177 |  |  |  |  | 1.17  (0.85-1.59) | 1.03  (0.76-1.39) |
|  |  |  |  |  |  |  | 18.5-24.9 | 1322 |  |  |  |  | 1^#^ | 1 |
|  |  |  |  |  |  |  | 25-29.9 | 513 |  |  |  |  | 1.18  (0.98-1.42) | 1.22  (1.01-1.48) |
|  |  |  |  |  |  |  | 30-34.9 | 265 |  |  |  |  | 1.32  (1.03-1.68) | 1.24  (0.95-1.61) |
|  |  |  |  |  |  |  | ≥35 | 179 |  |  |  |  | 1.92  (1.43-2.57) | 1.89  (1.39-2.58) |
|  |  |  |  |  |  |  | <18.5  (Black) | 79 |  |  |  |  | 1.52  (0.96-2.41) | 1.27  (0.81-1.99) |
|  |  |  |  |  |  |  | 18.5-24.9 | 533 |  |  |  |  | 1^#^ | 1 |
|  |  |  |  |  |  |  | 25-29.9 | 384 |  |  |  |  | 0.71  (0.53-0.96) | 0.79  (0.58-1.07) |
|  |  |  |  |  |  |  | 30-34.9 | 240 |  |  |  |  | 0.92  (0.66-1.29) | 0.96  (0.67-1.38) |
|  |  |  |  |  |  |  | ≥35 | 210 |  |  |  |  | 0.93  (0.65-1.34) | 1.02  (0.69-1.5) |
|  |  |  |  |  |  |  | <18.5  (White) | 302 | 0.55  (0.38-0.79) | 0.71  (0.47-1.05) |  |  |  |  |
|  |  |  |  |  |  |  | 18.5-24.9 | 1834 | 1^$^ | 1 |  |  |  |  |
|  |  |  |  |  |  |  | 25-29.9 | 721 | 0.79  (0.62-1.02) | 0.8  (0.61-1.04) |  |  |  |  |
|  |  |  |  |  |  |  | 30-34.9 | 385 | 0.72  (0.52-0.99) | 0.82  (0.57-1.16) |  |  |  |  |
|  |  |  |  |  |  |  | ≥35 | 275 | 0.56  (0.39-0.81) | 0.63  (0.42-0.94) |  |  |  |  |
|  |  |  |  |  |  |  | <18.5  (Black) | 192 | 0.84  (0.46-1.54) | 0.93  (0.5-1.74) |  |  |  |  |
|  |  |  |  |  |  |  | 18.5-24.9 | 1122 | 1^$^ | 1 |  |  |  |  |
|  |  |  |  |  |  |  | 25-29.9 | 715 | 1.17  (0.85-1.62) | 1.06  (0.75-1.48) |  |  |  |  |
|  |  |  |  |  |  |  | 30-34.9 | 444 | 1.4  (0.95-2.06) | 1.19  (0.77-1.48) |  |  |  |  |
|  |  |  |  |  |  |  | ≥35 | 367 | 1.34  (0.87-2.07) | 1.08  (0.68-1.7) |  |  |  |  |
| 15 | Cynthia J. Bartok [79] | United States | 2012 | prospective longitudinal cohort | 28.7±5.5 | self-reported | 18.5-24.9 | 399 |  |  |  |  |  | 1^#^ |
|  |  |  |  |  |  |  | 25-29.9 | 186 |  |  |  |  |  | 1.15  (0.88-1.51) |
|  |  |  |  |  |  |  | ≥30 | 133 |  |  |  |  |  | 1.43  (1.06-1.93) |
| 16 | Lindsay A. Thompson [36] | United States | 2013 | - | - | - | <18.5 | 62745 |  | 0.87  (0.85-0.89) |  |  |  |  |
|  |  |  |  |  |  |  | 18.5-24.9 | 592593 |  | 1^$^ |  |  |  |  |
|  |  |  |  |  |  |  | 25-29.9 | 267248 |  | 0.99  (0.97-1) |  |  |  |  |
|  |  |  |  |  |  |  | ≥30 | 220770 |  | 0.84  (0.83-0.85) |  |  |  |  |
| 17 | Gubler Tabea [38] | Swiss | 2013 | retrospective study | 30.79 ±5.56 | recorded | <18.5 | 134 |  | 1.61  (0.98-2.64) |  |  |  |  |
|  |  |  |  |  |  |  | 18.5-24.9 | 1288 |  | 1^**^ |  |  |  |  |
|  |  |  |  |  |  |  | 25-29.9 | 284 |  | 0.7  (0.51-0.98) |  |  |  |  |
|  |  |  |  |  |  |  | ≥30 | 114 |  | 0.3  (0.19-0.48) |  |  |  |  |
| 18 | Manal Dashti [80] | Kuwait | 2014 | prospective cohort | - | self-reported | <24.9 | 149 |  |  |  |  | 1^##^ |  |
|  |  |  |  |  |  |  | 25-29.9 | 124 |  |  |  |  | 1.03  (0.81-1.3) |  |
|  |  |  |  |  |  |  | ≥30 | 72 |  |  |  |  | 0.99  (0.75-1.31) |  |
|  |  |  |  |  |  |  | <24.9 | 149 |  |  |  |  | 1^###^ |  |
|  |  |  |  |  |  |  | 25-29.9 | 124 |  |  |  |  | 1.02  (0.72-1.45) |  |
|  |  |  |  |  |  |  | ≥30 | 72 |  |  |  |  | 1.21  (0.81-1.82) |  |
| 19 | Laura E Hauff [37] | United States | 2014 | longitudinal cohort study | - | self-reported | <25 | 1406 | 1^+^ | 1 |  |  |  |  |
|  |  |  |  |  |  |  | 25-29.9 | 741 | 1.05  (0.8-1.37) | 1.03  (0.66-1.59) |  |  |  |  |
|  |  |  |  |  |  |  | ≥30 | 677 | 0.77  (0.59-1) | 0.87  (0.57-1.33) |  |  |  |  |
|  |  |  |  |  |  |  | <25 | 1406 | 1^*^ | 1 |  |  |  |  |
|  |  |  |  |  |  |  | 25-29.9 | 741 | 1.08  (0.83-1.41) | 1.07  (0.63-1.84) |  |  |  |  |
|  |  |  |  |  |  |  | ≥30 | 677 | 0.76  (0.6-0.98) | 0.82  (0.49-1.38) |  |  |  |  |
|  |  |  |  |  |  |  | <25 | 1406 | 1^##^ | 1 |  |  |  |  |
|  |  |  |  |  |  |  | 25-29.9 | 741 | 1.12  (0.97-1.29) | 1.09  (0.93-1.27) |  |  |  |  |
|  |  |  |  |  |  |  | ≥30 | 677 | 1.32  (1.14-1.52) | 1.29  (1.09-1.53) |  |  |  |  |
|  |  |  |  |  |  |  | <25 | 1406 | 1^###^ | 1 |  |  |  |  |
|  |  |  |  |  |  |  | 25-29.9 | 741 | 1.14  (1.01-1.28) | 1  (0.87-1.15) |  |  |  |  |
|  |  |  |  |  |  |  | ≥30 | 677 | 1.16  (1.02-1.31) | 1.11  (0.96-1.29) |  |  |  |  |
| 20 | Saba W. Masho [70] | United States | 2015 | national surveillance | - | self-reported | <18.5 | 213108 | 1.42  (1.24-1.62) | 1.22  (1.07-1.4) |  |  |  |  |
|  |  |  |  |  |  |  | 18.5-24.9 | 2477498 | 1^$$^ | 1 |  |  |  |  |
|  |  |  |  |  |  |  | 25-29.9 | 1186841 | 1.14  (1.06-1.23) | 1.14  (1.06-1.23) |  |  |  |  |
|  |  |  |  |  |  |  | ≥30 | 1041886 | 1.46  (1.35-1.57) | 1.27  (1.18-1.37) |  |  |  |  |
| 21 | Julie Verret-Chalifour [71] | Canada | 2015 | prospective cohort study | - | self-reported | <18.5 | 337 |  |  | 1.19  (0.91-1.56) | 0.97  (0.75-1.25) |  |  |
|  |  |  |  |  |  |  | 18.5-24.9 | 4105 |  |  | 1^$$^ | 1 |  |  |
|  |  |  |  |  |  |  | 25-29.9 | 1317 |  |  | 1.15  (0.98-1.35) | 1.07  (0.91-1.24) |  |  |
|  |  |  |  |  |  |  | ≥30 | 833 |  |  | 1.69  (1.44-1.98) | 1.22  (1.04-1.42) |  |  |
| 22 | Kylee Cox [32] | Australia | 2015 | - | 30.2 ± 5.2 | - | <30  (at 26 week) | 290 |  |  |  |  |  | 1^**^ |
|  |  |  |  |  |  |  | ≥30 | 47 |  |  |  |  |  | 1.54  (1.04-2.3) |
|  |  |  |  |  |  |  | <30  (at 26 week) | 342 |  |  |  |  |  | 1^***^ |
|  |  |  |  |  |  |  | ≥30 | 64 |  |  |  |  |  | 2.83  (1.6-5) |
|  |  |  |  |  |  |  | <30  (at 52 week) | 290 |  |  |  |  |  | 1^***^ |
|  |  |  |  |  |  |  | ≥30 | 47 |  |  |  |  |  | 2.12  (1.33-3.4) |
| 23 | Josefa L. Martinez [23] | United States | 2016 | - | 25.6±5.5 | self-reported or measured | 25-29.9 | 238 | 1**^**^** | 1 |  |  |  |  |
|  |  |  |  |  |  |  | 30-34.9 | 132 | 1.8  (1.07-3.04) | 1.49  (0.79-2.79) |  |  |  |  |
|  |  |  |  |  |  |  | 35-39.9 | 64 | 3.26  (1.42-7.5) | 2.86  (1.1-7.39) |  |  |  |  |
|  |  |  |  |  |  |  | ≥40 | 46 | 2.67  (1.08-6.58) | 1.75  (0.61-5.07) |  |  |  |  |
| 24 | Contact H Castillo [72] | Brazil | 2016 | prospective cohort | 26.1 | extracted from prenatal card or when absent by self-report | 18.5-24.9 | 2286 |  |  |  |  |  | 1**^##^** |
|  |  |  |  |  |  |  | 25-29.9 | 877 |  |  |  |  |  | 1.49  (1.18-1.87) |
|  |  |  |  |  |  |  | ≥30 | 413 |  |  |  |  |  | 1.18  (1.02-1.37) |
| 25 | Susan J.de Jersey [7] | Australia | 2017 | prospective, observational study | 30±5 | self-reported | <25 | 267 |  | 1**^+^** |  |  |  |  |
|  |  |  |  |  |  |  | ≥25 | 135 |  | 0.57  (0.33-0.98) |  |  |  |  |
|  |  |  |  |  |  |  | <25  (at 4 mo) | 267 |  | 1**^+^** |  |  |  |  |
|  |  |  |  |  |  |  | ≥25 | 135 |  | 0.62  (0.4-0.97) |  |  |  |  |
| 26 | Naila Ramji [13] | Canada | 2017 | population-based, retrospective cohort | - | height and weight recorded | 18.5-24.9 | 5685 |  | 1**^$^** |  |  |  |  |
|  |  |  |  |  |  |  | 25-29.9 | 3394 |  | 0.86  (0.76-0.98) |  |  |  |  |
|  |  |  |  |  |  |  | 30-39.9 | 3394 |  | 0.65  (0.57-0.74) |  |  |  |  |
|  |  |  |  |  |  |  | 40-49.9 | 535 |  | 0.57  (0.44-0.74) |  |  |  |  |
|  |  |  |  |  |  |  | ≥50 | 71 |  | 0.37  (0.19-0.74) |  |  |  |  |
| 27 | Xing-Yong Tao [73] | China | 2017 | cohort | 26·4±3·6 | weight was self-reported/ height was measured | <18.5  (at 1 mo) | 605 |  |  | 1.02  (0.85-1.22) | 1.08  (0.9-1.3) |  |  |
|  |  |  |  |  |  |  | 18.5-23.9 | 2209 |  |  | 1^**^ | 1 |  |  |
|  |  |  |  |  |  |  | 24-27.9 | 300 |  |  | 1.08  (0.84-1.38) | 1.01  (0.78-1.29) |  |  |
|  |  |  |  |  |  |  | ≥28 | 82 |  |  | 1.62  (1.01-2.59) | 1.3  (0.8-2.12) |  |  |
|  |  |  |  |  |  |  | <18.5  (at 3 mo) | 605 |  |  | 1.03  (0.86-1.24) | 1.07  (0.88-1.29) |  |  |
|  |  |  |  |  |  |  | 18.5-23.9 | 2209 |  |  | 1^**^ | 1 |  |  |
|  |  |  |  |  |  |  | 24-27.9 | 300 |  |  | 1.2  (0.94-1.53) | 1.11  (0.86-1.43) |  |  |
|  |  |  |  |  |  |  | ≥28 | 82 |  |  | 1.35  (0.86-2.11) | 1.09  (0.68-1.75) |  |  |
|  |  |  |  |  |  |  | <18.5  (at 6 mo) | 605 |  |  | 0.77  (0.59-1.01) | 0.76  (0.58-1.01) |  |  |
|  |  |  |  |  |  |  | 18.5-23.9 | 2209 |  |  | 1^**^ | 1 |  |  |
|  |  |  |  |  |  |  | 24-27.9 | 300 |  |  | 0.86  (0.59-1.26) | 0.85  (0.57-1.25) |  |  |
|  |  |  |  |  |  |  | ≥28 | 82 |  |  | 0.73  (0.38-1.41) | 0.7  (0.36-1.37) |  |  |
| 28 | Afsaneh Tehranian [2] | Iran | 2017 | prospective cohort | 26.68 | self-report pre-pregnancy weight and height | <25 | 358 | 1**^#^** | 1 |  |  |  |  |
|  |  |  |  |  |  |  | ≥25 | 235 | 1.55  (0.8-2.88) | 2  (1.1-3.8) |  |  |  |  |
| 29 | Julie Boudet‐Berquier [74] | France | 2018 | prospective birth cohort | - | self‐reported | <18.5 | 161 |  |  | 0.97  (0.81-1.15) | 1.06  (0.91-1.23) |  |  |
|  |  |  |  |  |  |  | 18.5-24.9 | 1521 |  |  | 1**^###^** | 1**^###^** |  |  |
|  |  |  |  |  |  |  | 25-29.9 | 426 |  |  | 1.01  (0.9-1.13) | 1.01  (0.91-1.12) |  |  |
|  |  |  |  |  |  |  | ≥30 | 208 |  |  | 0.9  (0.76-1.08) | 0.86  (0.74-0.99) |  |  |
| 30 | Tanara Vogel Pinheiro [75] | Brazil | 2018 | prospective follow-up | 26.71 | the prenatal care booklet | 18.5-24.9 | 85 |  |  |  | 1**^$$^** |  |  |
|  |  |  |  |  |  |  | ≥25 | 64 |  |  |  | 1.05  (0.99-1.1) |  |  |
| 31 | Nicole E Marshall [41] | United States | 2018 | cohort longitudinal study | 32.8±4.6  34.5±4.8  30.4±7.8  for each category | self-reported | 18.5-24.9  (at 6 week) | 58 |  | 1**^**^** |  |  |  |  |
|  |  |  |  |  |  |  | ≥25 | 51 |  | 0.24  (0.08-0.75) |  |  |  |  |
|  |  |  |  |  |  |  | 18.5-24.9  (at 6 mo) | 49 |  | 1 |  |  |  |  |
|  |  |  |  |  |  |  | ≥25 | 22 |  | 0.17  (0.07-0.41) |  |  |  |  |
| 32 | Tammy Campbell [40] | United Kingdom | 2018 | cohort study | - | self-reported | <18.5 | 999 | 0.63  (0.54-0.74) |  |  |  |  |  |
|  |  |  |  |  |  |  | 18.5-24.9 | 11266 | 1^$^ |  |  |  |  |  |
|  |  |  |  |  |  |  | 25-29.9 | 3373 | 0.86  (0.77-0.96) |  |  |  |  |  |
|  |  |  |  |  |  |  | ≥30 | 1475 | 0.83  (0.72-0.96) |  |  |  |  |  |
|  |  |  |  |  |  |  | <18.5  (at first week) |  | 1.32  (1.05-1.66) |  |  |  |  |  |
|  |  |  |  |  |  |  | 18.5-24.9 |  | 1^*^ |  |  |  |  |  |
|  |  |  |  |  |  |  | 25-29.9 |  | 1.59  (1.38-1.83) |  |  |  |  |  |
|  |  |  |  |  |  |  | ≥30 |  | 1.63  (1.35-1.97) |  |  |  |  |  |
|  |  |  |  |  |  |  | <18.5  (for 4 mo) |  | 0.79  (0.65-0.96) |  |  |  |  |  |
|  |  |  |  |  |  |  | 18.5-24.9 |  | 1^*^ |  |  |  |  |  |
|  |  |  |  |  |  |  | 25-29.9 |  | 0.75  (0.67-0.84) |  |  |  |  |  |
|  |  |  |  |  |  |  | ≥30 |  | 0.58  (0.48-0.69) |  |  |  |  |  |

**Uncited references in the text**

[63] J.L. Baker, K.F. Michaelsen, K.M. Rasmussen, Sorensen TI, Maternal prepregnant

body mass index, duration of breastfeeding, and timing of complementary food

introduction are associated with infant weight gain, Am. J. Clin. Nutr. 80 (6)

(2004) 1579–1588.

[64] A.M. Grjibovski, A. Yngve, L.O. Bygren, M. Sjostrom, Socio-demographic

determinants of initiation and duration of breastfeeding in northwest Russia, Acta

Paediatr. 94 (5) (2005) 588–594.

[65] W.H. Oddy, J. Li, L. Landsborough, G.E. Kendall, S. Henderson, J. Downie, The

association of maternal overweight and obesity with breastfeeding duration,

J. Pediatr. 149 (2) (2006) 185–191.

[66] J.L. Baker, K.F. Michaelsen, T.I. Sorensen, K.M. Rasmussen, High prepregnant

body mass index is associated with early termination of full and any breastfeeding

in Danish women, Am. J. Clin. Nutr. 86 (2) (2007) 404–411.

[67] S.M. Donath, L.H. Amir, Maternal obesity and initiation and duration of

breastfeeding: data from the longitudinal study of Australian children, Matern.

Child Nutr. 4 (3) (2008) 163–170.

[68] P. Kitsantas, L.R. Pawloski, Maternal obesity, health status during pregnancy, and

breastfeeding initiation and duration, the journal of maternal-fetal & neonatal

medicine: the official journal of the European Association of Perinatal Medicine,

the Federation of Asia and Oceania Perinatal Societies, Int. Soc. Perinatal Obstet

23 (2) (2010) 135–141.

[69] S.T. Natland, L.F. Andersen, T.I. Nilsen, S. Forsmo, G.W. Jacobsen, Maternal recall

of breastfeeding duration twenty years after delivery, BMC Med. Res. Methodol.

12 (2012) 179.

[70] S.W. Masho, S. Cha, M.R. Morris, Prepregnancy obesity and breastfeeding

noninitiation in the United States: an examination of racial and ethnic differences,

Breastfeed. Med.: Off. J. Acad. Breastfeed. Med. 10 (5) (2015) 253–262.

[71] J. Verret-Chalifour, Y. Giguère, J.C. Forest, J. Croteau, P. Zhang, I. Marc,

Breastfeeding initiation: impact of obesity in a large Canadian perinatal cohort

study, PloS One 10 (2) (2015).

[72] H. Castillo, I.S. Santos, A. Matijasevich, Maternal pre-pregnancy BMI, gestational

weight gain and breastfeeding, Eur. J. Clin. Nutr. 70 (4) (2016) 431–436.

[73] X.Y. Tao, K. Huang, S.Q. Yan, A.Z. Zuo, R.W. Tao, H. Cao. Pre-pregnancy BMI,

gestational weight gain and breast-feeding: a cohort study in China,

Public health nutrition 20 (6) (2017) 1001-8.

[74] J. Boudet-Berquier, B. Salanave, J.C. Desenclos, K. Castetbon, Association between

maternal prepregnancy obesity and breastfeeding duration: data from a

nationwide prospective birth cohort, Matern. Child Nutr. 14 (2) (2018).

[75] T.V. Pinheiro, M.Z. Goldani, Maternal pre-pregnancy overweight/obesity and

gestational diabetes interaction on delayed breastfeeding initiation, PloS One 13

(6) (2018).

[76] I.H. Rutishauser, J.B. Carlin, Body mass index and duration of breast feeding: a

survival analysis during the first six months of life, J. Epidemiol. Community

Health 46 (6) (1992) 559–565.

[77] Y. Manios, E. Grammatikaki, K. Kondaki, E. Ioannou, A. Anastasiadou, M. Birbilis,

The effect of maternal obesity on initiation and duration of breast-feeding in

Greece: the GENESIS study, Publ. Health Nutr. 12 (4) (2009) 517–524.

[78] H.L. Kehler, K.H. Chaput, S.C. Tough, Risk factors for cessation of breastfeeding

prior to six months postpartum among a community sample of women in Calgary,

Alberta, Canad. J. Publ. Health/Revue Canadienne de Sante’e Publique (2009) 376–380.

[79] C.J. Bartok, E.W. Schaefer, J.S. Beiler, I.M. Paul, Role of body mass index and

gestational weight gain in breastfeeding outcomes, Breastfeed. Med.: Off. J. Acad.

Breastfeed. Med. 7 (6) (2012) 448–456.

[80] M. Dashti, J.A. Scott, C.A. Edwards, M. Al-Sughayer, Predictors of breastfeeding

duration among women in Kuwait: results of a prospective cohort study, Nutrients

6 (2) (2014) 711–728
